# Glucocerebrosidase deficiency promotes protein aggregation through dysregulation of extracellular vesicles

**DOI:** 10.1371/journal.pgen.1007694

**Published:** 2018-09-26

**Authors:** Ruth E. Thomas, Evelyn S. Vincow, Gennifer E. Merrihew, Michael J. MacCoss, Marie Y. Davis, Leo J. Pallanck

**Affiliations:** 1 Department of Genome Sciences, University of Washington, Seattle, WA, United States of America; 2 Department of Neurology, University of Washington, Seattle, WA, United States of America; 3 Department of Neurology, Veterans Affairs Puget Sound Health Care System, Seattle, WA, United States of America; University of Pennsylvania, UNITED STATES

## Abstract

Mutations in the *glucosylceramidase beta* (*GBA*) gene are strongly associated with neurodegenerative diseases marked by protein aggregation. *GBA* encodes the lysosomal enzyme glucocerebrosidase, which breaks down glucosylceramide. A common explanation for the link between *GBA* mutations and protein aggregation is that lysosomal accumulation of glucosylceramide causes impaired autophagy. We tested this hypothesis directly by measuring protein turnover and abundance in *Drosophila* mutants with deletions in the *GBA* ortholog *Gba1b*. Proteomic analyses revealed that known autophagy substrates, which had severely impaired turnover in autophagy-deficient *Atg7* mutants, showed little to no overall slowing of turnover or increase in abundance in *Gba1b* mutants. Likewise, *Gba1b* mutants did not have the marked impairment of mitochondrial protein turnover seen in mitophagy-deficient *parkin* mutants. Proteasome activity, microautophagy, and endocytic degradation also appeared unaffected in *Gba1b* mutants. However, we found striking changes in the turnover and abundance of proteins associated with extracellular vesicles (EVs), which have been proposed as vehicles for the spread of protein aggregates in neurodegenerative disease. These changes were specific to *Gba1b* mutants and did not represent an acceleration of normal aging. Western blotting of isolated EVs confirmed the increased abundance of EV proteins in *Gba1b* mutants, and nanoparticle tracking analysis revealed that *Gba1b* mutants had six times as many EVs as controls. Genetic perturbations of EV production in *Gba1b* mutants suppressed protein aggregation, demonstrating that the increase in EV abundance contributed to the accumulation of protein aggregates. Together, our findings indicate that glucocerebrosidase deficiency causes pathogenic changes in EV metabolism and may promote the spread of protein aggregates through extracellular vesicles.

## Author summary

Mutations in the *GBA* gene, which encodes the enzyme glucocerebrosidase, are common and increase the risk of Parkinson disease. A widely accepted explanation for the increased risk is that the fatty substance normally broken down by glucocerebrosidase builds up in the lysosome, which is the cell’s recycling center, until the cell can no longer get rid of damaged parts. At that point, proteins that should be destroyed in the lysosome form large clumps (aggregates) throughout the cell. We used mutant fruit flies without glucocerebrosidase to test this theory, and we were surprised to see no evidence that the lysosome was failing. The destruction of proteins usually recycled by the lysosome was not slowed down in the mutant flies. Instead, we saw evidence that the mutants’ cells might be producing too many extracellular vesicles, tiny spheres that transport cargo and messages from cell to cell. Some researchers have also suggested that extracellular vesicles carry the protein aggregates that spread between cells as Parkinson disease get worse. Our study supports this idea. It suggests that increased spread of aggregates through extracellular vesicles, rather than failure of the lysosome, might explain why *GBA* mutations increase the risk of neurodegenerative disease.

## Introduction

Mutations in the gene encoding the lysosomal enzyme glucocerebrosidase, *glucosylceramidase beta* (*GBA*), are associated with neurodegeneration and brain protein aggregation [1, 2]. Homozygous mutations in *GBA* cause the lysosomal storage disorder Gaucher disease, which in some cases includes devastating neurological symptoms [3], while heterozygous *GBA* mutations are the strongest risk factor for both Parkinson disease (PD) and the related disorder dementia with Lewy bodies [1, 2, 4]. Up to 10% of individuals with nonfamilial PD carry a *GBA* mutation [5]. In addition, PD patients with a *GBA* mutation have faster progression of both motor and cognitive symptoms [6]. To study the mechanisms underlying the association between *GBA* mutations and neurodegeneration, we created a *Drosophila* model of glucocerebrosidase (GCase) deficiency. *Drosophila* has two *GBA* homologs, designated *Gba1a* and *Gba1b*. The *Gba1a* gene is expressed exclusively in the midgut [7], and deletion of this gene does not appear to confer deleterious phenotypes [8]. By contrast, the *Gba1b* gene is ubiquitously expressed [7], and *Gba1b* deletion causes marked abnormalities. We previously reported that *Gba1b* null mutants exhibit phenotypes including shortened lifespan, locomotor and memory deficits, neurodegeneration, accumulation of the autophagy adaptor Ref(2)P (p62/SQSTM1), and accumulation of ubiquitinated protein aggregates [9]. Similar phenotypes were subsequently seen in an independently generated *Gba1b* null mutant [8].

The protein aggregation and elevated Ref(2)P levels in *Gba1b* mutants suggested that they had impaired autophagy, as did morphological changes in the autolysosomal system noted by Kinghorn et al. [8, 9]. These findings are consistent with previous reports of autolysosomal impairment upon loss of GCase activity [1, 10–15]. Based on such findings, we and others hypothesized that lysosomal accumulation of glucosylceramide, the normal substrate of GCase, leads to impairment of autophagy [12, 16–18]. However, none of the work implicating autophagy in the pathogenic effects of GCase deficiency has yet established that GCase loss of function causes global impairment of autophagic degradation.

To investigate the autophagy failure model of *GBA* pathogenesis, we used proteomics-based techniques to measure protein turnover and abundance in *Gba1b* mutants and controls, as well as in flies with mutations in key autophagy (*Atg7*) or mitophagy (*parkin*) genes [19, 20]. While *Atg7* mutants showed marked and widespread slowing of autophagy substrate turnover, *Gba1b* mutants did not. The effects of *Gba1b* mutation on the turnover and abundance of autophagy substrates also failed to correlate with those of *Atg7* or *parkin* mutations. Moreover, we detected no deficits in turnover mediated by the proteasome, microautophagy, or endocytosis. However, we found high incidences of faster turnover and increased abundance among proteins associated with extracellular vesicles (EVs), which have been previously suggested as a mechanism for the spread of protein aggregates in neurodegenerative disease. Biochemical studies confirmed increased abundance of EV marker proteins in isolated EVs from *Gba1b* mutants, and nanoparticle tracking analysis showed that the mutants had markedly increased numbers of EVs. Genetic manipulations to reduce EV production decreased the accumulation of ubiquitinated protein aggregates and Ref(2)P in *Gba1b* mutants, supporting the model that excessive EV abundance promotes the accumulation of protein aggregates. Together, our findings suggest that the most important pathological consequence of *Gba1b* loss of function is not failure of autophagic protein degradation but excessive production of extracellular vesicles.

## Results

### *Gba1b* mutations do not cause global impairment of autophagic protein degradation

To test the hypothesis that GCase deficiency causes impaired autophagic turnover, we compared protein degradation rates in heads from *Gba1b* mutants and controls using stable isotope labeling. In brief, our method involves feeding flies a stable heavy isotope of leucine and then using mass spectrometry to monitor the rate at which unlabeled proteins are degraded and replaced with labeled proteins [21]. We measured the influence of *Gba1b* loss of function on all proteins with data that met quality standards in both *Gba1b* mutants and controls (1297 proteins for turnover analysis, 4221 for abundance; S1 Data). We analyzed turnover data with Topograph [22], software specifically designed for measurement of protein turnover via stable isotope labeling. We also compared protein abundance in *Gba1b* and control flies using Skyline [23] and MSstats [24]. Fold change in turnover and fold change in abundance were then calculated for every protein. Fold change for a protein was calculated as the value in *Gba1b* mutants divided by the value in controls.

**Fig 1:**
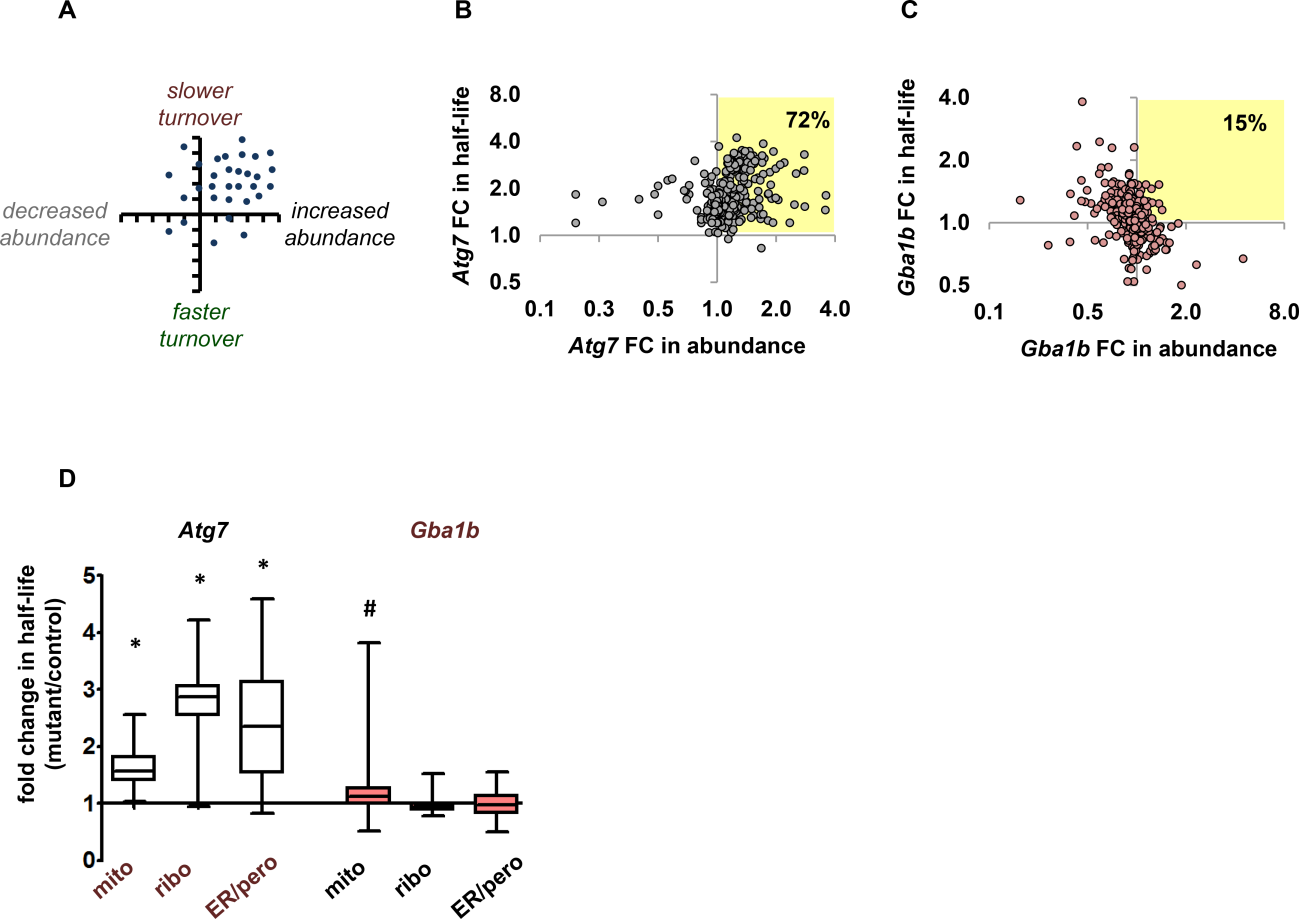
*Gba1b* mutants do not have changes in protein turnover or abundance consistent with impaired autophagy. (A) Predicted effects of impaired autophagy on turnover and abundance of autophagy substrates. Targets of autophagy should have slower turnover (fold change in half-life >1) and increased or unchanged abundance. Datapoints represent theoretical individual proteins. (B) Pattern of turnover and abundance change in heads from a *Drosophila* autophagy mutant (*Atg7* null). Fold change (FC) in half-life vs. fold change in abundance for proteins from known organellar targets of autophagy (mitochondria, cytosolic ribosomes, endoplasmic reticulum (ER), and peroxisomes; *n* = 295). As predicted, the vast majority of these proteins (72%) have slower turnover and either increased or unchanged abundance (highlighted quadrant). Fold change = mutant half-life or abundance value divided by corresponding control value. (C) Turnover change vs. abundance change in heads from *Drosophila Gba1b* mutants. Only 15% of autophagy substrate proteins match the autophagy mutant pattern of slower turnover and increased or unchanged abundance. (D) Autophagy substrate proteins have dramatically slower mean turnover in *Atg7* mutants, but not in *Gba1b* mutants. Box plot shows fold change in half-life (box: median and quartiles; whiskers: maximum and minimum values). Half-lives of mitochondrial, ribosomal, and ER/peroxisomal proteins (*n* = 186, 53, and 32 respectively) were significantly longer in *Atg7* mutants compared to controls (*p < 0.001 by nested ANOVA). ER and peroxisomes were combined for analysis due to the low numbers of peroxisomal proteins detected. In *Gba1b* mutants, only mitochondrial proteins (*n* = 258) had significantly slower mean turnover compared to controls (^#^*p* = 0.02 by nested ANOVA; *n* = 63 ribosomal proteins and 51 ER/peroxisomal proteins).

We predicted that autophagy substrates would show slower turnover (longer half-lives) in *Gba1b* mutants, and that they might show increased abundance if synthesis did not decrease to match the slower degradation rate (Fig 1A). We defined autophagy substrates as proteins from mitochondria, cytosolic ribosomes, endoplasmic reticulum (ER), and peroxisomes, all previously identified as targets of autophagy [25–30]. We validated our prediction using turnover and abundance data from autophagy-deficient *Atg7* mutants, which we characterized in previous work [21] (S1 Data). We first plotted *Atg7* fold change in turnover against fold change in abundance for autophagy substrates to observe the overall pattern of proteostasis changes (Fig 1B). *Atg7* mutants showed changes consistent with our prediction: the vast majority of autophagy substrates (72%) had slower turnover (fold change in half-life >1) and increased or unchanged abundance. We therefore used *Atg7* mutant data as a reference for the effects of autophagy impairment. When we plotted fold change in turnover against fold change in abundance for *Gba1b* mutants, the pattern of changes was markedly different; only 15% of autophagy substrate proteins had slower turnover and increased or unchanged abundance (Fig 1C). Proteostasis of autophagy substrates in *Gba1b* mutants thus did not overall resemble the pattern seen in *Atg7* mutants.

To compare in more detail the effects of *Gba1b* and *Atg7* mutations on protein turnover and abundance, we performed several additional analyses, beginning by calculating *Gba1b* and *Atg7* mean fold change in turnover (half-life) for the autophagy substrate proteins mentioned above. Each mutant was compared to its own control. Turnover of proteins from all three classes of autophagy substrates was significantly slowed in *Atg7* mutants (*p* < 0.001 by nested ANOVA), but in *Gba1b* mutants there was no overall change in the half-lives of ribosomal or ER/peroxisomal proteins (Fig 1D; *p* = NS by nested ANOVA) and only a very mild slowing of mean mitochondrial protein turnover (mean fold change 1.15 ± 0.32; *p* = 0.02 by nested ANOVA; Fig 1D).

**Fig 2:**
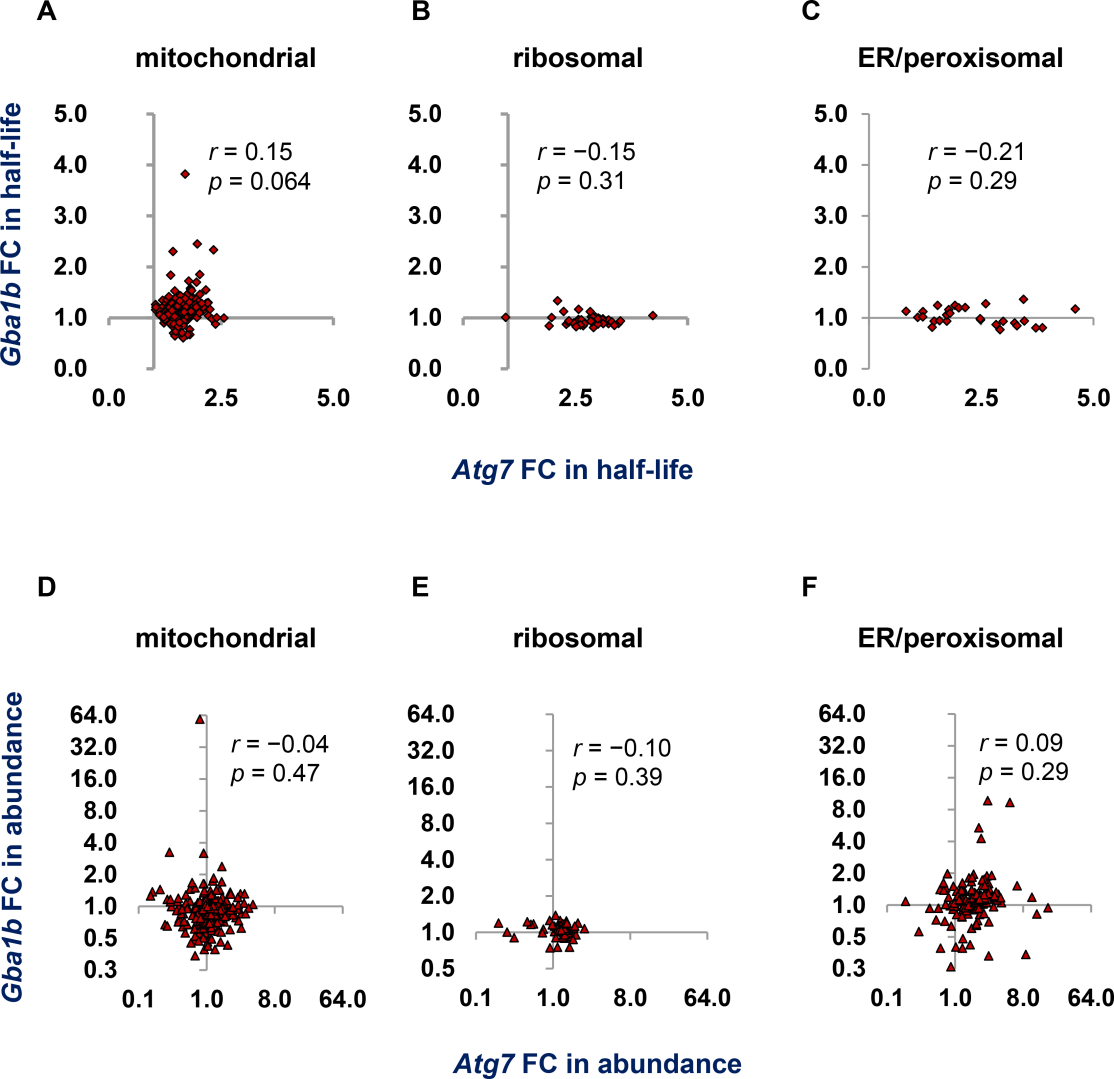
Protein-by-protein comparison reveals no relationship between the effects of *Gba1b* mutations and *Atg7* mutations on proteostasis. (A-C) Correlation between *Gba1b* and *Atg7* fold changes (FC) in half-life for the following groups of proteins common to both fly head datasets: (A) Mitochondrial proteins (*n* = 161). (B) Proteins of the cytosolic ribosome (*n* = 49). (C) Proteins of the endoplasmic reticulum and peroxisome (*n* = 28). Fold change was calculated for each mutant compared to its own control strain. (D-F) Correlation between *Gba1b* and *Atg7* fold changes in abundance for the following groups of proteins common to both datasets: (D) Mitochondrial proteins (*n* = 394). (E) Ribosomal proteins (*n* = 76). (F) ER and peroxisomal proteins (*n* = 131).

To test further for evidence of impaired autophagy in *Gba1b* mutants, we compared the effects of *Atg7* and *Gba1b* mutations on individual proteins. We began with turnover, plotting the fold change in half-life for *Gba1b* mutants (*Gba1b* mutant half-life/*Gba1b* control half-life) against the fold change for *Atg7* mutants (*Atg7* mutant/*Atg7* control). We compared *Gba1b* and *Atg7* effects on individual proteins from each of the three autophagy substrate categories. There was no statistically significant relationship between the effects of *Gba1b* and those of autophagy ablation for mitochondrial, ribosomal, or ER/peroxisomal proteins (Fig 2A–2C). We also tested for a relationship between *Gba1b* and *Atg7* effects on protein abundance (Fig 2D–2F) and found no significant correlation for any of the three autophagy substrate groups. The effects of *Gba1b* loss of function on protein turnover and abundance thus do not resemble the effects of autophagy ablation, and we find no evidence that *Gba1b* mutation causes global impairment of autophagic protein degradation.

### *Gba1b* mutations do not cause impairment of mitophagy

**Fig 3:**
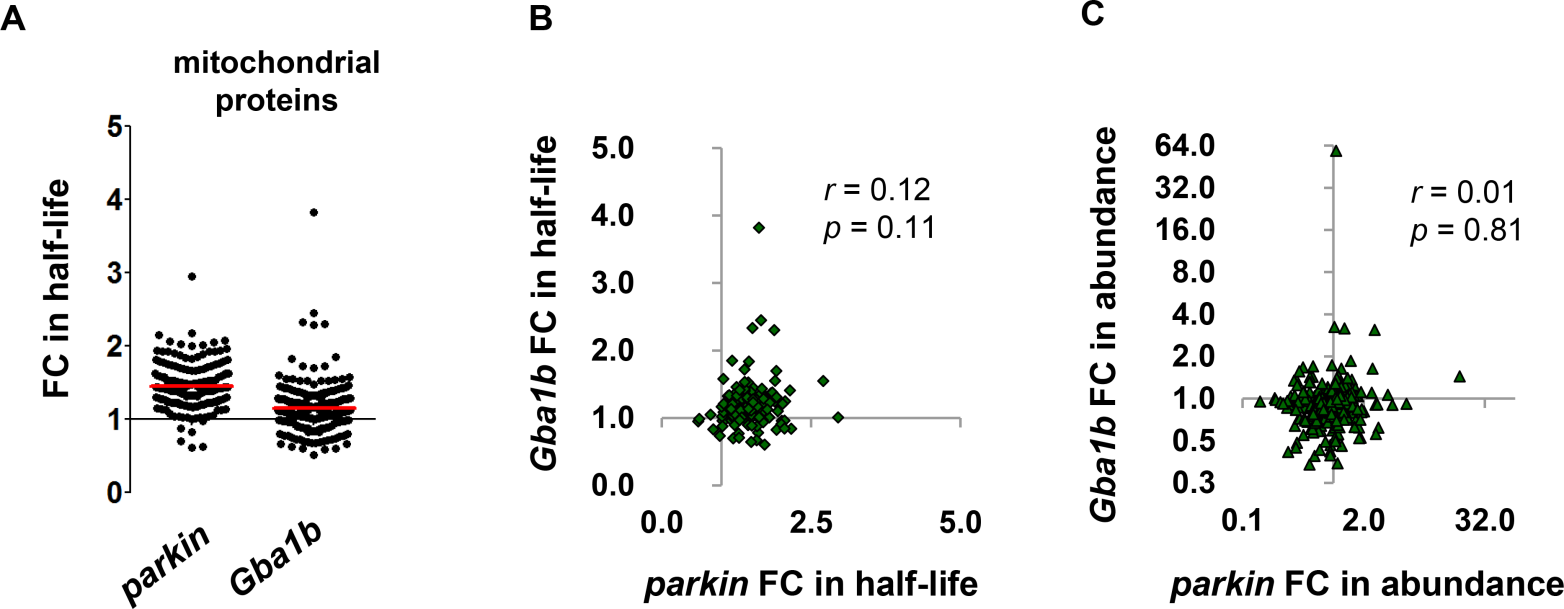
Comparison to *parkin* mutants reveals no evidence of impaired mitophagy in *Gba1b* mutants. (A) Fold change (FC) in half-life values for fly head mitochondrial proteins in *parkin* and *Gba1b* mutants compared to their respective controls. Each dot represents one protein; *n* = 179 for *parkin*, 258 for *Gba1b*. The red line represents the mean. Mean fold change for *parkin* was 1.45 ± 0.31, and mean fold change for *Gba1b* 1.15 ± 0.32 (significantly slower by *t* test, *p* < 0.0001). (B) Correlation between *Gba1b* and *parkin* fold changes in half-life for mitochondrial proteins (*n* = 167 common to both datasets). (C) Correlation between *Gba1b* and *parkin* fold changes in abundance for mitochondrial proteins (*n* = 395).

One reported consequence of *GBA* loss of function is accumulation of dysfunctional mitochondria due to defective mitophagy [31, 32]; the slight but statistically significant slowdown of mitochondrial protein turnover in *Gba1b* mutants therefore raised the possibility of a mild mitophagy deficit. We had previously found a mitochondrial protein turnover deficit in flies with mutations in the mitophagy factor *parkin* [21], and we now compared the effects of *Gba1b* mutation on mitochondrial proteostasis with those of *parkin*. In *parkin* mutants, turnover was slowed for the vast majority of mitochondrial proteins (Fig 3A). In *Gba1b* mutants, changes in mitochondrial protein turnover were both milder and less consistent (Fig 3A, S1 Data). We considered the possibility that *Gba1b* mutants had a mitophagy defect that was obscured by compensatory upregulation of other mitochondrial protein turnover mechanisms, as we previously found in *PINK1*^*B9*^ mutants, which lack a mitophagy factor upstream of Parkin [21]. In *PINK1*^*B9*^ mutants, while the mean fold change in mitochondrial protein half-life was not significantly altered, the effects of *PINK1*^*B9*^ mutation on individual proteins correlated strongly with those of *parkin* mutation. We therefore tested whether the effect of *Gba1b* on mitochondrial proteostasis would also correlate with the effect of *parkin*. However, we detected no significant correlation between *Gba1b* and *parkin* effects on mitochondrial protein turnover (Fig 3B) or abundance (Fig 3C). Our findings therefore do not support either globally impaired autophagy or selectively impaired mitophagy in *Gba1b* mutants.

### *Gba1b* mutants have no evidence of impairment in other protein degradation systems

**Fig 4:**
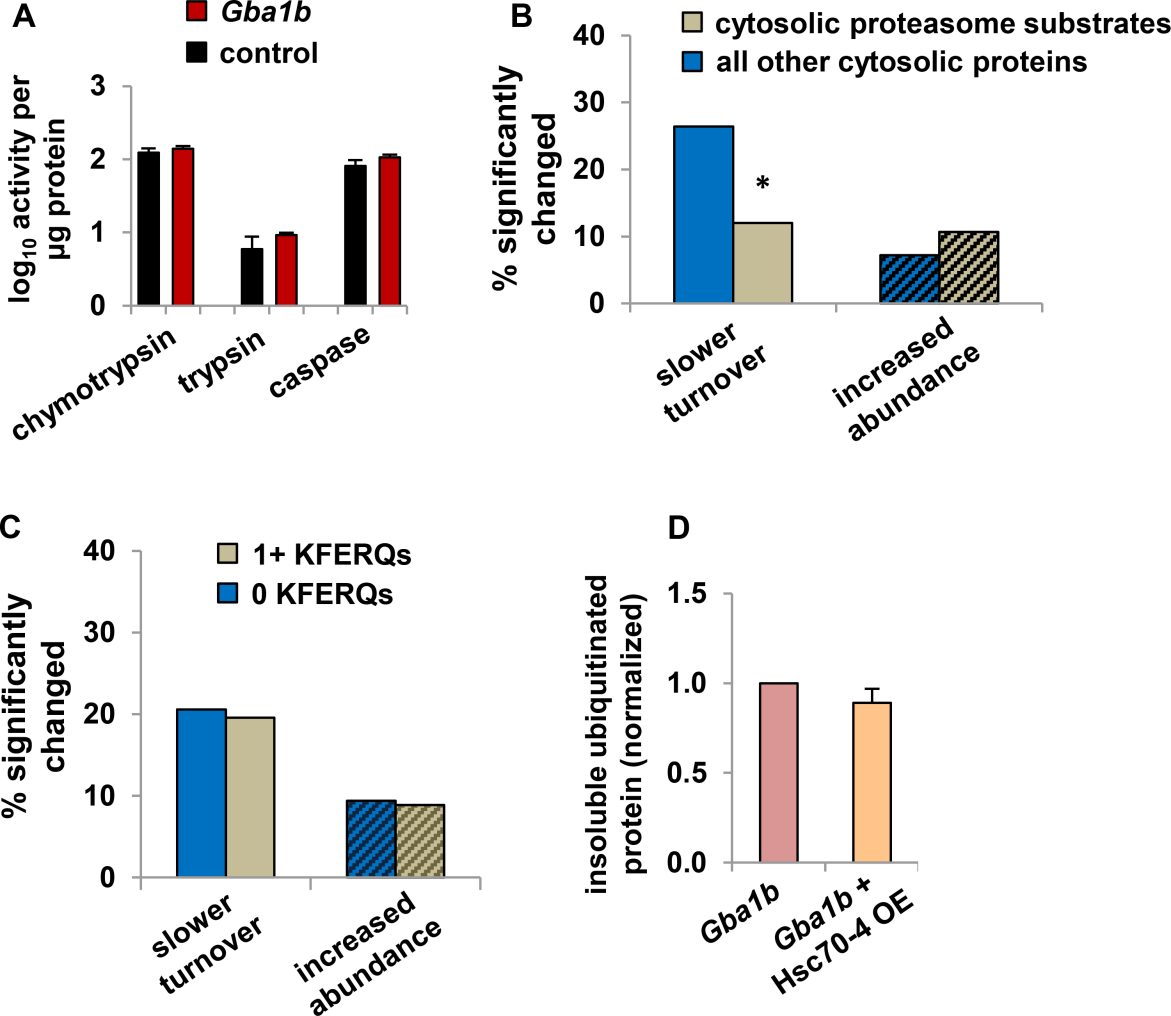
Proteasome activity and endosomal microautophagy are unaffected in *Gba1b* mutants. (A) Measurement of proteasome activity from fly heads using fluorescent substrates, normalized to total protein, in *Gba1b* mutants and controls. Nonproteasomal (epoxomicin-insensitive) background activity was subtracted. Error bars represent SEM. (B) Percentage of cytosolic proteasome substrates (see Materials and Methods) with significantly slower turnover or increased abundance in heads from *Gba1b* mutants, compared to all other cytosolic proteins. Turnover: *n* = 100 substrate and 144 other proteins. Proteasome substrates are less likely than other proteins in the dataset to have slowed turnover (*p = 0.0062 by Fisher exact test). Abundance: *n* = 178 substrate and 375 other proteins; there was no significant difference between substrates and other proteins in the frequency of increased abundance by Fisher exact test. Solid bars indicate turnover and bars with diagonal lines indicate abundance. (C) Percentage of microautophagy substrate proteins with slower turnover or increased abundance in *Gba1b* mutants. There is no significant difference between cytosolic proteins with and without KFERQ-like microautophagy targeting sequences (Fisher exact test). For turnover, *n* = 138 proteins with and 107 without KFERQs; for abundance, *n* = 319 and 233. (D) Insoluble ubiquitinated protein in heads from 10-day-old *Gba1b* mutants with and without overexpression (OE) of Hsc70-4, measured by western blotting. Ubiquitin signal was normalized to Actin, and then expressed as a proportion of the normalized signal in sibling controls. There was no significant difference between genotypes by Student *t* test (*p* = 0.33). Error bars represent SEM. The results of three independent experiments are shown.

The lack of evidence for autophagy failure led us to consider alternative explanations for the accumulation of ubiquitin-positive protein aggregates in *Gba1b* mutants. We first considered the possibility that these aggregates could arise because of reduced proteasome function, which is known to lead to the formation of large ubiquitin-positive protein aggregates called aggresomes [33]. We tested proteasome activity using fluorescent substrates, and found that all three enzyme activities were normal (Fig 4A). We also considered the possibility that delivery of substrates to the proteasome might be impaired [34], and used our proteomic data to determine whether actual proteasome substrates were degraded normally in *Gba1b* mutants. We identified cytosolic proteasome substrates based on data from Wagner et al. [35] (S2 Data) and compared turnover and abundance changes in this group of proteins to the changes in all other cytosolic proteins. If proteasomal degradation were impaired, we would expect substrates of the process to have slowed turnover and possibly increased abundance, as we predicted for autophagy impairment. In fact, however, the percentage of proteins with slowed turnover in *Gba1b* mutants was significantly lower for proteasome substrates than for other cytosolic proteins, and proteasome substrates did not show a greater incidence of increased abundance in *Gba1b* mutants (Fig 4B). Together, these results indicate that proteasome dysfunction does not underlie the accumulation of ubiquitin-positive aggregates in *Gba1b* mutants.

We next examined whether the protein aggregation in *Gba1b* mutants could be the result of altered endosomal functioning. As *Gba1b* mutants have markedly increased levels of glucosylceramide (S1 Fig, [8]) and moderately decreased levels of ceramide (S1 Fig), abnormal membrane composition could compromise functioning of the endosomal system [36, 37]. We therefore tested for impairment of endosomal microautophagy, an Hsc70-4–dependent process that degrades cytosolic proteins with specific targeting sequences (“KFERQ-like motifs”) [38]. To test whether microautophagy is impaired in *Gba1b* mutants, we searched the *Drosophila* proteome for proteins with KFERQ-like motifs, and compared the effects of *Gba1b* on cytosolic proteins with and without such motifs. Compared to proteins without KFERQ-like motifs, proteins with one or more KFERQ-like motifs did not have an increased incidence of proteins with slower turnover or increased abundance (Fig 4C, S2 Data). We also tested whether overexpression of Hsc70-4, which has been shown to increase microautophagy in *Drosophila* [39], would influence the accumulation of insoluble ubiquitinated protein. However, this manipulation had no effect on the abundance of ubiquitinated protein aggregates (Fig 4D). We thus found no evidence that impaired endosomal microautophagy is responsible for the accumulation of ubiquitinated protein aggregates in *Gba1b* mutants.

**Fig 5:**
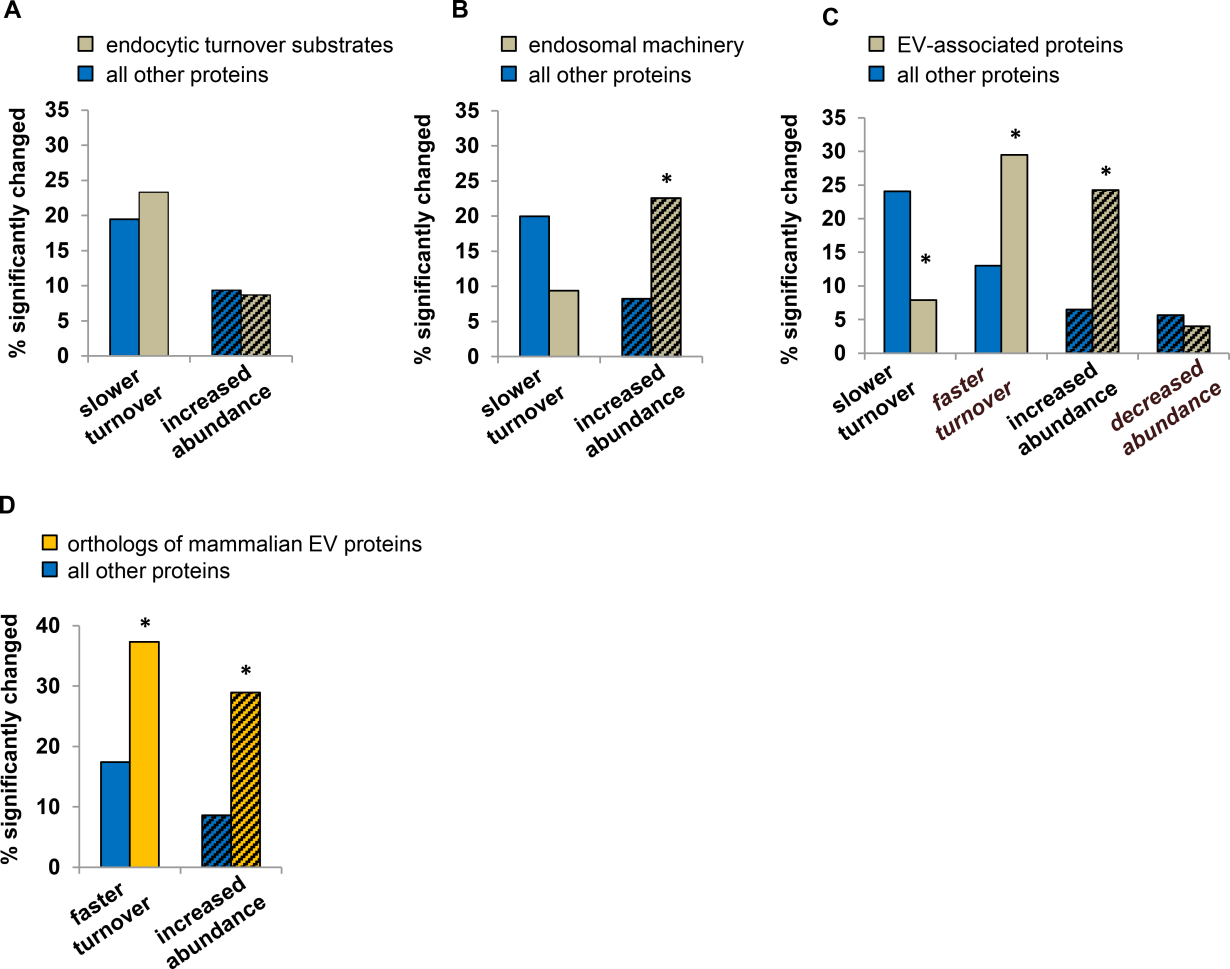
Endocytic degradation is normal in *Gba1b* mutants, but extracellular vesicle proteins show altered turnover and abundance. (A) Percentage of endocytic turnover substrates and all other proteins in the dataset that have significantly slowed turnover or increased abundance in heads from *Gba1b* mutants. There was no significant difference between substrates and other proteins in either turnover or abundance in *Gba1b* mutants. Turnover: *n* = 90 endocytic turnover substrates, 1207 other proteins; *p* = 0.41 by Fisher exact test. Abundance: *n* = 437 substrates, 3784 other proteins; *p* = 0.99 by χ^2^. Solid bars indicate turnover and bars with diagonal lines indicate abundance. (B) Percentage of endosomal machinery proteins with significantly slowed turnover or increased abundance in *Gba1b* mutants. Endosomal machinery refers to proteins that reside in endosomes or take part in endocytosis (*n* = 32 for turnover, 102 for abundance). Endosomal machinery proteins are not included in the list of endocytic turnover substrates. The percentage of proteins with significantly slowed turnover was not different for endosomal machinery proteins than for the remaining proteins (*p* = 0.18 by Fisher exact test). The percentage of proteins with increased abundance, however, was greater in endosomal machinery proteins than in all other proteins (*p < 0.0001 by χ^2^ test). (C) Turnover and abundance changes in proteins associated with extracellular vesicles (EVs; *n* = 329 EV proteins and 968 other proteins for turnover, 499 EV proteins and 3722 other proteins for abundance). Proteins were identified as EV-associated using a compiled list of *Drosophila* EV proteins (see Materials and Methods). Compared to non-EV proteins, a smaller percentage of EV-associated proteins had slowed turnover (*p < 0.0001 by Fisher exact test), and larger percentages of EV-associated proteins had accelerated turnover (*p < 0.0001 by Fisher exact test) and increased abundance (*p < 0.0001 by χ^2^ test). (D) *Drosophila* orthologs of the ExoCarta “top 100” list of proteins most frequently detected in mammalian EVs (*n* = 59 for turnover, 83 for abundance). Faster turnover and increased abundance both occurred more frequently in these EV-associated proteins than in all other proteins. *p < 0.0001 by Fisher exact test (turnover) and χ^2^ (abundance).

We then investigated whether *Gba1b* mutations impaired the functioning of another endosomal degradation pathway, endocytic turnover. Using FlyBase [40] and other annotation resources, we identified typical substrates of this pathway, primarily integral cell membrane proteins (*n* = 90 in turnover data, 437 in abundance data; S2 Data). We also identified a separate group of “endosomal machinery” proteins, which reside in endosomes or are required for endocytosis (*n* = 32 in turnover data, 102 in abundance data; S2 Data). We found no evidence that degradation of endocytic turnover substrates was compromised; compared to all other proteins, endocytic turnover substrates did not have a higher frequency of significantly slowed turnover or increased abundance (Fig 5A). When we examined endosomal machinery, however, we found a higher prevalence of proteins with increased abundance (*p* < 0.0001 vs. all other proteins by Fisher exact test; Fig 5B). Thus, *Gba1b* mutants had no evidence of compromised endocytic turnover, but proteostasis of the endosomal machinery was clearly altered.

### Proteostasis of extracellular vesicle proteins is altered in *Gba1b* mutants

Many endosomal machinery proteins also play roles in the creation and release of extracellular vesicles (EVs), a heterogeneous population of membrane-delimited structures originating from the multivesicular endosome and plasma membrane [41, 42]. EVs transport varied cargoes of protein and nucleic acids from cell to cell and play roles in signaling, waste disposal, and intercellular resource transfer [43–45]. EVs have also been implicated in the spread of protein aggregates in neurodegenerative disease [41]. Given that *Gba1b* mutants have altered turnover and abundance of endosomal machinery proteins but not endocytic turnover substrates, we considered the alternative possibility that GCase deficiency influences EV biology. To explore this hypothesis, we first tested whether proteins known to be associated with EVs showed significant alterations in turnover or abundance in *Gba1b* mutants. We compiled a list of proteins detected in EVs from *Drosophila* cultured cells [46–49]; the resulting list contained 544 nonredundant proteins (S3 Data), 329 of which were found in the *Gba1b* turnover data and 499 in the abundance data. Compared to all other proteins in the dataset, a smaller percentage of EV-associated proteins had slowed turnover, and a higher percentage had faster-than-normal turnover (*p* < 0.0001 by Fisher exact test; Fig 5C). In addition, a greater proportion of EV proteins had increased abundance in *Gba1b* mutants (*p* < 0.0001 by Fisher exact test; Fig 5C). To confirm that EV-associated proteins had faster turnover and increased abundance in *Gba1b* mutants, we repeated our analysis using an independent list of EV proteins. We obtained the ExoCarta [47] “top 100” list of proteins most frequently identified in mammalian EVs and identified their *Drosophila* orthologs using DIOPT v6.0 [50] (*n* = 97; S3 Data). Once again, compared to the rest of the dataset, EV-associated proteins had higher frequencies of faster turnover and increased abundance in *Gba1b* mutants (Fig 5D), suggesting that GCase deficiency may cause dysregulation of EV biology.

### Changes in EV proteostasis are specific to *Gba1b* mutants

**Fig 6:**
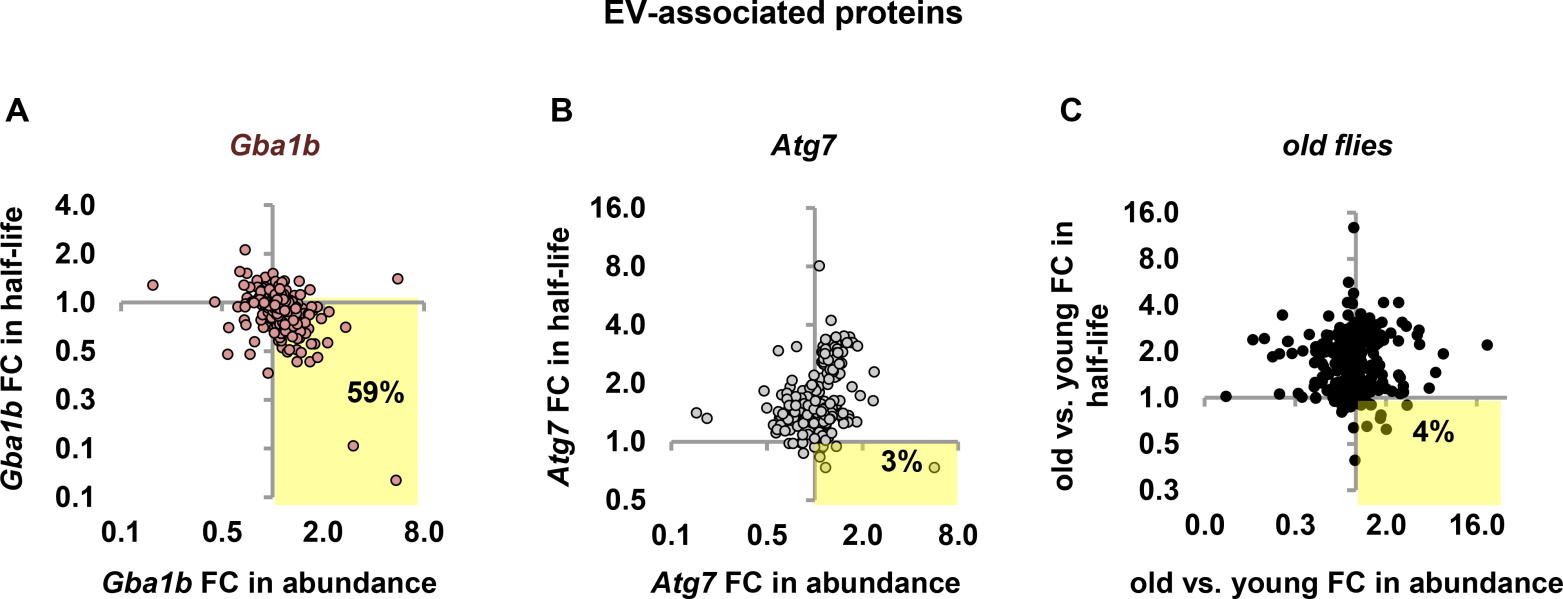
EV proteostasis changes are specific to *Gba1b* mutants. (A-C) Turnover change vs. abundance change for EV-associated proteins in (A) *Gba1b* mutants (*n* = 314 proteins), (B) *Atg7* mutants (*n* = 236), and (C) old vs. young flies (*n* = 299). As before, all measurements were performed on fly head extracts. Old flies were 55–60 days old at the start of labeling. Highlighted quadrant indicates EV proteins with faster turnover and increased abundance. FC = fold change.

To test whether faster turnover and increased abundance of EV-associated proteins are specifically associated with *Gba1b* loss of function, we investigated whether these proteins were also disproportionately affected by other conditions that alter protein turnover. We evaluated the pattern of changes, as we had done for autophagy substrates, by plotting fold change in turnover against fold change in abundance for all EV-associated proteins. In *Gba1b* mutants, 59% of the datapoints representing EV proteins appeared in the quadrant representing faster turnover and increased abundance (Fig 6A); in *Atg7* mutants, only 3% of EV-associated proteins showed the same pattern (Fig 6B). We also looked at the pattern of EV proteostasis in other mutants described in our previous work [21]: the mitophagy mutants *parkin* and *PINK1*, and the oxidative stress mutant *Sod2*. Because abundance data for these mutants lacked enough significant changes for analysis, we analyzed turnover only. None of these mutants showed faster turnover of EV proteins (S2 Fig).

We also investigated whether the EV proteostasis alterations in *Gba1b* mutants represented a distinctive pathological process or simply an acceleration of normal aging, given that ubiquitinated protein aggregates accumulate with age even in wild-type flies [51–53]. To do this, we measured protein turnover and abundance in old flies (55 to 60 days at the start of labeling) and young flies (5 days). Old flies had dramatically slower turnover of most proteins (mean fold change in half-life for all proteins 2.46 ± 4.31) and milder changes in protein abundance (both increases and decreases; S4 Data). In old flies, only 4% of EV-associated proteins were represented by datapoints in the faster turnover/increased abundance quadrant (Fig 6C), indicating that the altered EV proteostasis observed in *Gba1b* mutants does not represent an acceleration of normal aging. Together, our findings indicate that altered proteostasis of EV-associated proteins is a specific and novel feature of *Gba1b* mutants.

### EV-associated proteins are more abundant in EVs from *Gba1b* mutants

**Fig 7:**
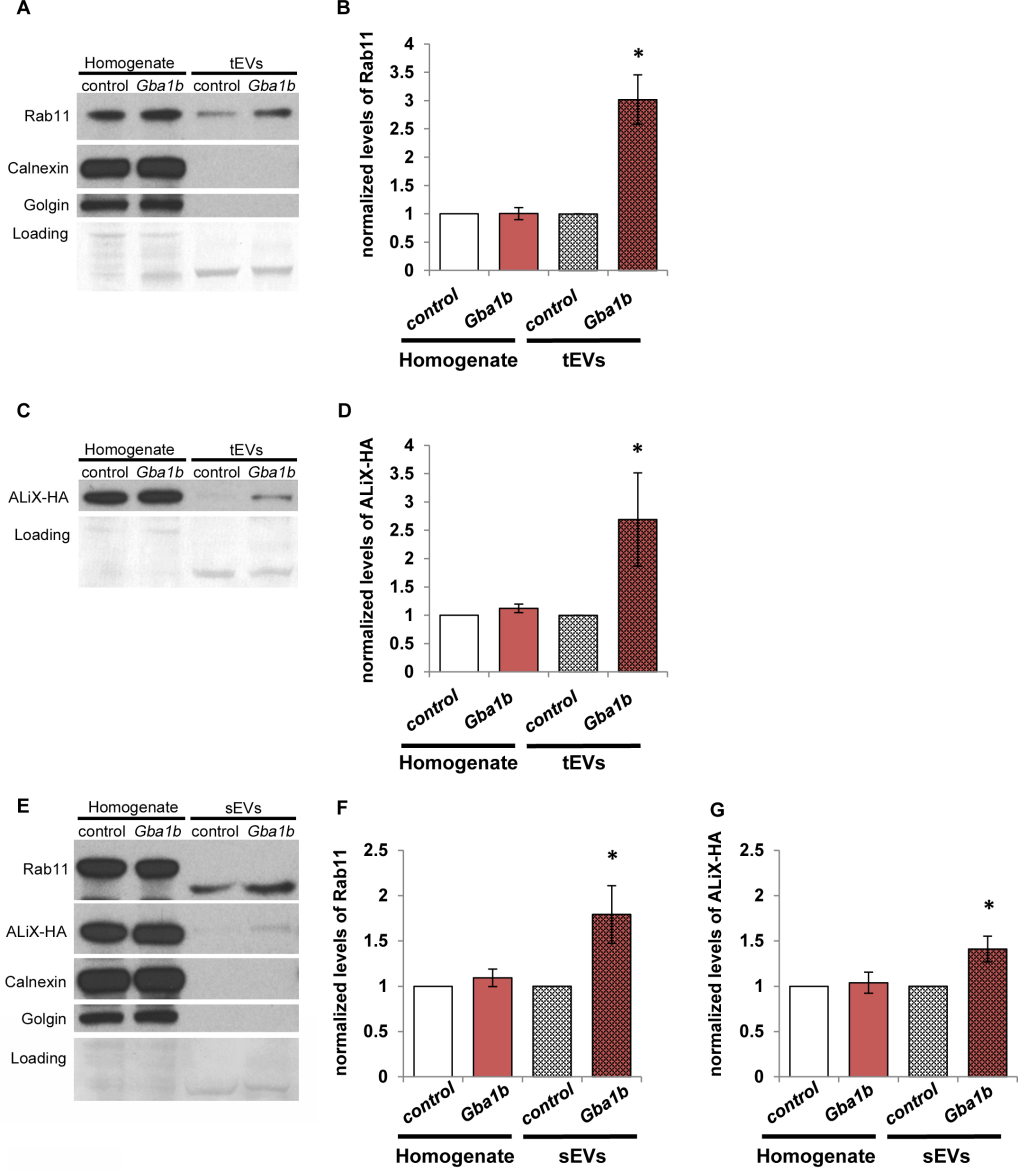
EV marker proteins are more abundant in isolated EVs of *Gba1b* mutants. (A) Whole-fly homogenates and total extracellular vesicles (tEVs) from *Gba1b* mutants and controls were probed with an antibody to Rab11. The blots were also probed with antibodies to microsomal markers Calnexin (Cnx99A) and Golgin (Golgin84) to demonstrate the purity of the EV samples. (B) Quantification of Rab11 in tEVs. Homogenate signal was normalized to Ponceau-S loading; all EV samples were normalized to loading volume. Mutant values were then normalized to corresponding control values. *Gba1b* = *Gba1b*^*ΔTT*^*/Gba1b*^*ΔTT*^; control = *Gba1b*^*rv*^*/ Gba1b*^*rv*^. (C) Pan-neuronal driver *elav-GAL4* was used to express ALiX-HA in *Gba1b* mutants and controls. Whole-fly homogenates and tEVs from these flies were probed with an antibody to HA. *Gba1b* = *Gba1b*^*ΔTT*^*/Gba1b*^*MB03039*^; control = *Gba1b*^*rv*^*/Gba1b*^*MB03039*^. (D) Quantification of ALiX-HA in tEVs. Normalization was performed as in panel B. (E) Whole-fly homogenates and isolated small extracellular vesicles (sEVs; see Materials and Methods) were probed with antibodies to Rab11, HA, Calnexin, and Golgin. (F) Quantification of Rab11 in sEVs. (G) Quantification of ALiX-HA in sEVs. At least three independent experiments were performed. Representative images are shown. Error bars represent SEM. *p < 0.05 by Student *t* test.

As mentioned above, all of our proteomic analyses were performed using protein extracts from fly heads. To test whether the observed alterations in EV protein abundance were also evident in EVs themselves, we performed western blotting for known EV markers on EV fractions from hemolymph, the *Drosophila* equivalent of blood. To do this, we collected cell-free hemolymph extracts containing the full range of circulating EVs, which we designated total EVs (tEVs). We also prepared extracts containing only EVs under 220 nm in size, which we designated small EVs (sEVs). We then performed western blot analysis on tEVs or sEVs compared to whole-fly homogenate to measure the abundance of two EV marker proteins: Rab11 and an HA-tagged form of ALiX (PDCD6IP) [48, 54]. We also used western blotting to verify EV isolation by the absence of microsomal markers Calnexin (Cnx99A) and Golgin (Golgin84; Fig 7A and 7E) according to International Society for Extracellular Vesicles standards [55].

Rab11 and ALiX-HA were significantly increased in abundance in *Gba1b* mutants vs. controls in both tEVs and sEVs, but not in whole-fly homogenate (Fig 7A–7E). Although the Rab11 detected in sEVs was 3–5 kDa smaller than in the whole-fly homogenate, this finding is consistent with previous work demonstrating altered molecular weights for several proteins when detected in EVs [56]. A GFP-tagged form of Rab11 also showed increased abundance in sEVs from *Gba1b* mutants (S3 Fig). The findings using tagged forms of EV proteins are particularly informative because these exogenous proteins were expressed at equivalent overall levels in controls and *Gba1b* mutants (Fig 7G, S3 Fig). The increased abundance of these markers in EVs from *Gba1b* mutants indicates that either more of each marker protein is loaded into each EV, or that *Gba1b* mutants produce more EVs.

### Ref(2)P is present in *Drosophila* EVs and is more abundant in EVs from *Gba1b* mutants

**Fig 8:**
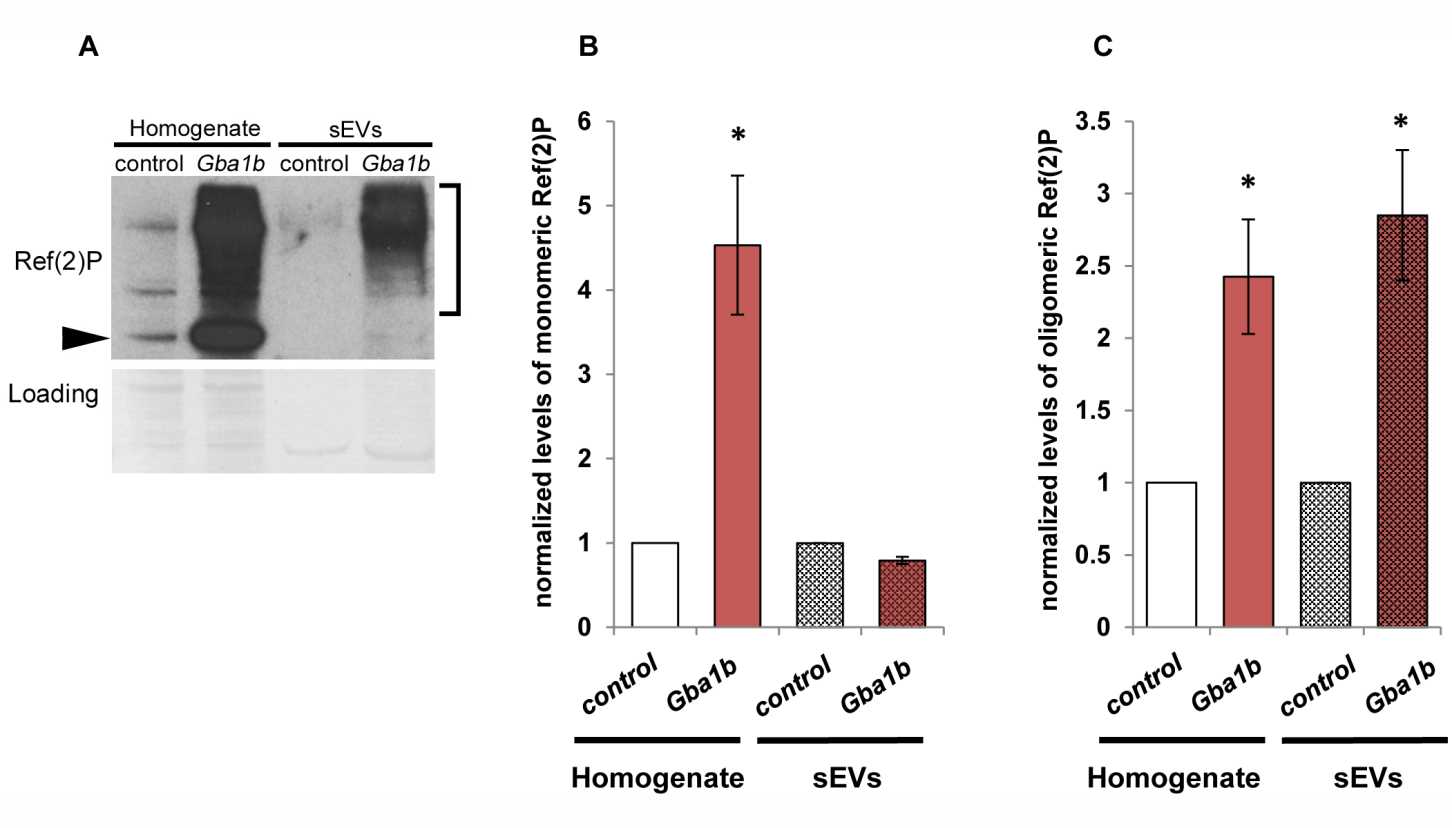
High molecular weight Ref(2)P is more abundant in *Gba1b* mutant EVs. Whole-fly homogenates and isolated extracellular vesicle extracts from *Gba1b* mutants and controls were subjected to western blot analysis using an antibody to Ref(2)P. *Gba1b* = *Gba1b*^*ΔTT*^*/Gba1b*^*ΔTT*^; control = *Gba1b*^*rv*^*/ Gba1b*^*rv*^. (A) Arrowhead indicates monomeric Ref(2)P; bracket indicates high molecular weight Ref(2)P. (B) Quantification of monomeric Ref(2)P. (C) Quantification of high molecular weight Ref(2)P. At least three independent experiments were performed. Representative image is shown. Error bars represent SEM. *p < 0.01 by Student *t* test.

One of the most striking abnormalities in *Gba1b* mutants is their accumulation of Ref(2)P [9], the *Drosophila* p62 ortholog, which was markedly elevated by proteomic measurement (S1 Data). This is especially noteworthy given that accumulation of Ref(2)P is usually interpreted as an indication of impaired autophagic flux [57–59], and yet we find no evidence of impaired autophagic degradation in *Gba1b* mutants. Ref(2)P/p62 has multiple functions, however, and mammalian p62 has been detected in EVs [47, 60]. We therefore performed western blotting for Ref(2)P on sEVs from *Gba1b* mutants and controls to test whether Ref(2)P accumulates in EVs. The sEVs contained very little monomeric Ref(2)P, but did reveal a marked increase in higher molecular weight Ref(2)P oligomers (Fig 8A–8C), which were approximately three times as abundant in *Gba1b* mutants as in controls (Fig 8C). We confirmed that these high molecular weight bands represented Ref(2)P by performing RNAi knockdown of *Ref(2)P* in *Gba1b* mutants (S4 Fig). The increased Ref(2)P abundance in *Gba1b* mutant EVs suggests that changes in EVs may contribute to the markedly increased Ref(2)P seen in *Gba1b* mutant heads.

### *Gba1b* mutants have markedly increased numbers of EVs

**Fig 9:**
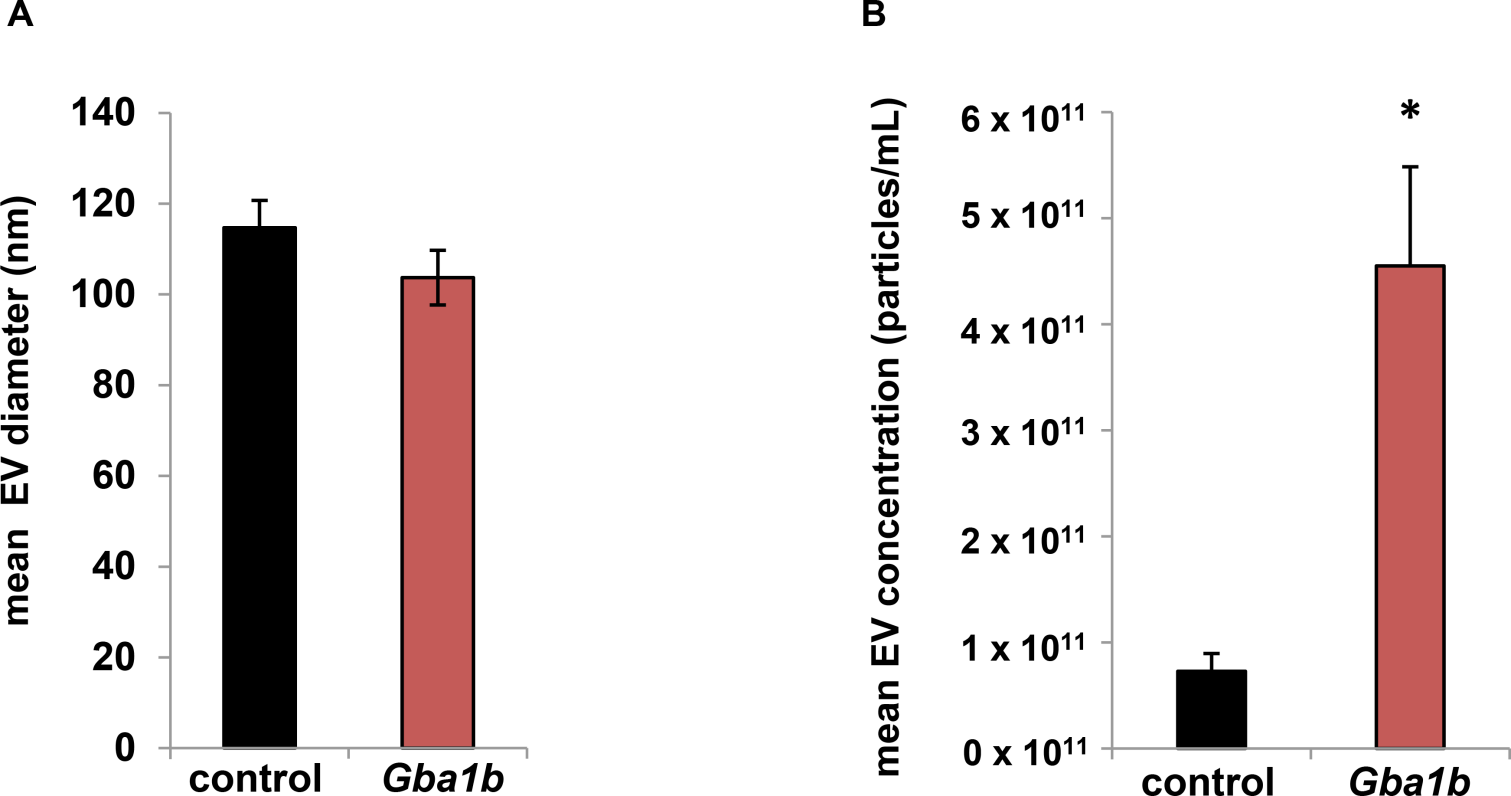
Nanoparticle tracking analysis reveals that *Gba1b* mutants have a sixfold increase in EVs compared to controls. Size and concentration of hemolymph-derived EVs as measured by nanoparticle tracking analysis using a ZetaView instrument and software version 8.04.02. (A) Average EV diameter in *Gba1b* mutants (103.7 ± 12.1 nm) and controls (114.7 ± 12.1 nm). (B) Average EV concentration in *Gba1b* mutants (4.55 x 10^11^ ± 1.87 x 10^11^ particles/mL) and controls (7.28 x 10^10^ ± 3.36 x 10^10^ particles/mL). Four biological replicates per genotype were analyzed. Error bars represent SEM. *p < 0.05 by Student *t* test.

As mentioned above, the increased abundance of multiple EV-associated proteins in *Gba1b* mutants suggests either that more of each protein is loaded into each EV, or that more EVs are produced. To distinguish these possibilities, we performed nanoparticle tracking analysis on EVs from the hemolymph of *Gba1b* mutants and controls. For these experiments, we chose to use a 0.65 μm rather than a 0.22 μm filter to retain EVs of as many sizes as possible while still ensuring removal of all cell debris. While the mean size of EVs was comparable in *Gba1b* mutants and controls (Fig 9A), the concentration of EVs was approximately six times higher in the mutants (Fig 9B). The mean concentrations were 4.55 x 10^11^ particles/mL (± 1.87 x 10^11^) for *Gba1b* mutants and 7.28 x 10^10^ particles/mL (±3.36 x 10^10^) for controls (*p* = 0.013 by Student *t* test). Thus, the increased abundance of EV proteins in *Gba1b* mutants is best explained by the increased production of EVs. Together, our findings give clear evidence of altered EV biology in *Gba1b* mutants.

### Reducing ESCRT-dependent EV release decreases protein aggregation in *Gba1b* mutants

**Fig 10:**
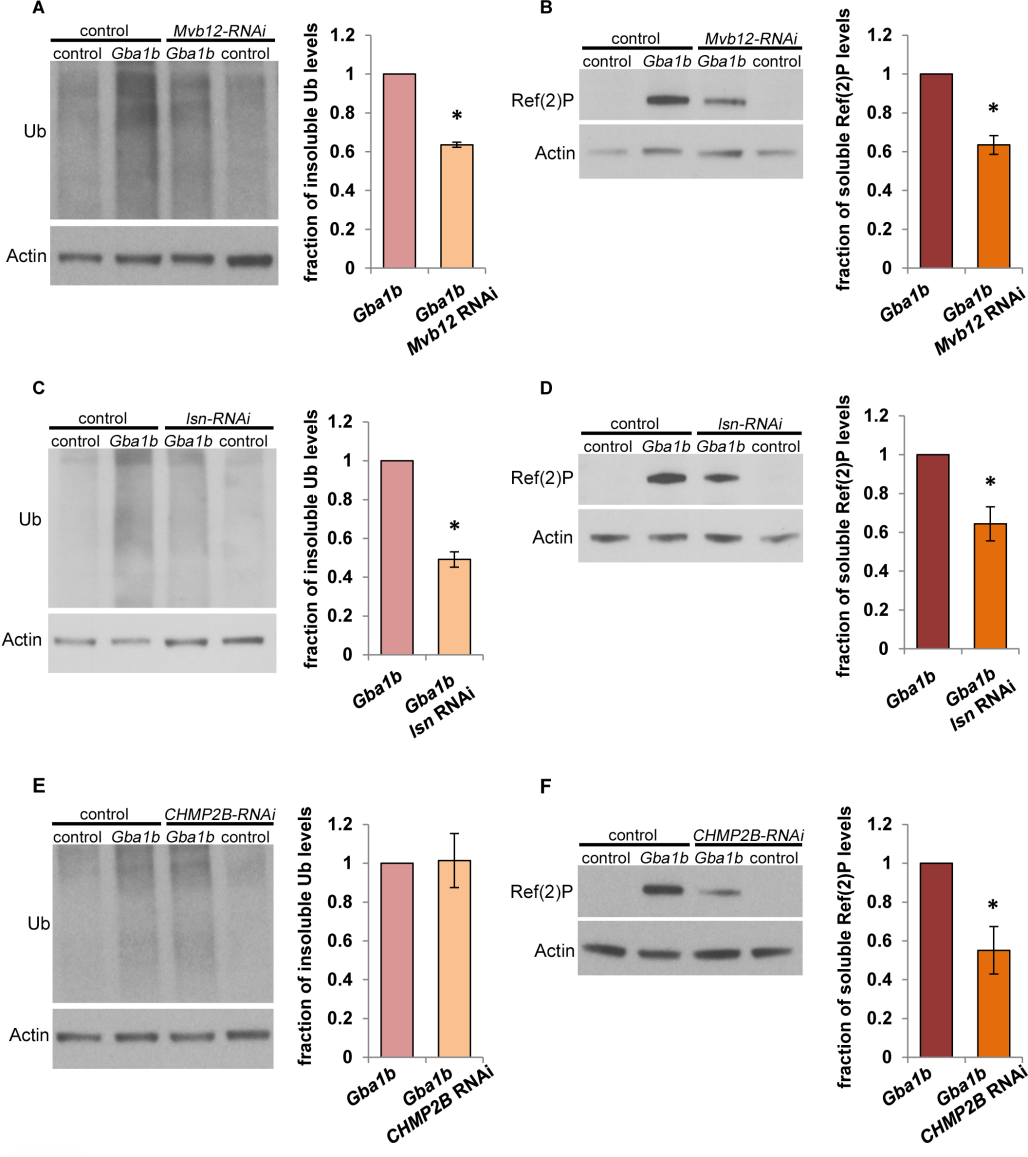
Knockdown of ESCRT proteins suppresses the accumulation of ubiquitinated protein aggregates and Ref(2)P in *Gba1b* mutants. (A-F) RNAi constructs were expressed using the pan-neuronal driver *elav-GAL4* in *Gba1b* mutants (*Gba1b*^*ΔTT*^*/Gba1b*^*ΔTT*^*)* and controls (*Gba1b*^*rv*^*/ Gba1b*^*rv*^). Homogenates were prepared from fly heads using 1% Triton X-100. Western blot analysis was performed on the Triton X-100–insoluble proteins using antibodies to ubiquitin (Ub) and Actin, and on the soluble fractions using antibodies to Ref(2)P and Actin. (A-B) Representative images and quantification of (A) ubiquitin and (B) Ref(2)P from flies with and without *Mvb12* knockdown. (C-D) Representative images and quantification of (C) ubiquitin and (D) Ref(2)P from flies with and without knockdown of *lsn*. (E-F) Representative images and quantification of (E) ubiquitin and (F) Ref(2)P from flies with and without knockdown of *CHMP2B*. At least three independent experiments were performed. Error bars represent SEM. *p < 0.05 by Student *t* test.

As previously noted, EVs have been repeatedly described as possible vehicles for the spread of brain protein aggregation in neurodegenerative disease [41]. Our finding that *Gba1b* mutants had more EVs led us to hypothesize that increased EV release promotes protein aggregation by increasing cell-to-cell transmission of aggregation-prone proteins. As a first step toward testing this model, we determined whether the accumulation of protein aggregates in *Gba1b* mutants could be suppressed by knocking down components of the ESCRT (endosomal sorting complexes required for transport) pathway, which are required for production of many types of EVs [41, 54]. Using a pan-neuronal driver, we expressed RNAi against proteins from three of the four ESCRT complexes: *Mvb12* (*Multivesicular body subunit 12*; ESCRT-I), *lsn* (*larsen/Vps22*; ESCRT-II), and *CHMP2B* (*Charged multivesicular body protein 2b;* ESCRT-III). We found that knockdown of each of the three ESCRT proteins significantly reduced accumulation of Ref(2)P in *Gba1b* mutants, and that knockdown of *Mvb12* and *lsn* also reduced the accumulation of insoluble ubiquitinated protein (Fig 10A–10F). These findings support the model that excessive production of EVs is responsible for the accumulation of protein aggregates caused by GCase deficiency.

## Discussion

Impairment of autolysosomal degradation is widely thought to explain the increased risk of neurodegeneration associated with mutations in *GBA*, which encodes the lysosomal enzyme glucocerebrosidase (GCase) [1, 16], and multiple studies have found hallmarks of impaired autophagy associated with GCase loss of function. These hallmarks have included accumulation of ubiquitinated protein aggregates, increased abundance of autophagic flux markers such as p62/SQSTM1 and LC3-II, impairment of autophagosome-lysosome fusion, and changes in the size and number of autophagosomes and lysosomes [12, 13, 61–66]. These indications that GCase deficiency leads to autophagy impairment have been found in diverse experimental systems, including multiple animal models, cultured cells, iPSC-derived human neuronal models, and postmortem patient samples [8, 11–13, 31, 66–70]. Our own initial characterization of *Drosophila Gba1b* mutants, which revealed extensive ubiquitinated protein aggregates and markedly elevated levels of the p62 ortholog Ref(2)P, also appeared to support the model that GCase deficiency impairs autophagic degradation [9]. In our current work, however, proteomic measurement of protein turnover and abundance showed no evidence that degradation of autophagy substrates was globally impaired in *Gba1b* mutants. The mutants also showed no evidence of failure in other protein degradation pathways. Instead, we found faster turnover and increased abundance of proteins associated with extracellular vesicles (EVs). Followup experiments on isolated EVs confirmed increased abundance of EV marker proteins and revealed a strikingly increased number of EVs. Furthermore, genetic manipulations that reduced EV formation suppressed both the increased protein aggregation and the increased Ref(2)P abundance observed in *Gba1b* mutants. Our findings suggest that dysregulation of extracellular vesicles, rather than failure of autophagic degradation, may be the primary mechanism by which GCase deficiency leads to protein aggregation and neurodegeneration.

Although the many previous reports of autophagy impairment in GCase-deficient organisms appear incompatible with our current protein turnover findings, we do not believe that our findings contradict previous work. When we measure common markers of autolysosomal function such as Ref(2)P/p62 and insoluble ubiquitinated protein, *Drosophila Gba1b* mutants show results comparable to those seen in vertebrate models of GCase deficiency [10, 15, 68, 69, 71]. Our proteomic measurements of protein abundance are also consistent with previous reports of increased lysosomal mass in GCase deficiency [1, 8, 66]. The abundance of the lysosomal marker Lamp1 was nearly tripled in *Gba1b* mutants, and 41% of lysosomal proteins were significantly increased in abundance (S1 Data). Nevertheless, our protein turnover measurements reveal that the overall rates of degradation through lysosomal processes are not grossly altered. Thus, one possible explanation of our findings is that the efficiency of autolysosomal degradation is decreased, with lower throughput per unit of autolysosomal mass, but that the organism has compensated by increasing the amount of autolysosomal machinery available. Because this compensation is sufficient to maintain degradation rates, we would describe *Gba1b* mutants as being under autolysosomal stress rather than in autolysosomal failure. Over time, the degree of stress may exceed the capacity to compensate, and aged *Gba1b* mutants may show overt failure of lysosomal degradation. Even if this is the case, late failure of autolysosomal degradation cannot explain the behavioral and biochemical abnormalities that begin in early adulthood [8, 9].

Another explanation for the apparent discrepancy between our findings of normal autophagic substrate turnover and previous reports of impaired autophagy is that commonly used autophagy markers are not solely representative of autophagic flux [57, 72]. This is especially true of Ref(2)P, or p62, which has multiple nonautophagic functions and is transcriptionally upregulated by stress [57, 73]. In addition, p62 and LC3 have recently been detected in mammalian EVs [47, 74, 75], and we found increased levels of oligomeric Ref(2)P in EVs from *Gba1b* mutants (Fig 8). It is therefore possible that the increased Ref(2)P levels detected in *Gba1b* mutants result from a combination of stress response and EV dysregulation.

Our work leaves unanswered the question of how GCase deficiency results in increased EV abundance, but does suggest two possible explanations. Increased production of EVs could be caused either by lysosomal stress or by changes in membrane lipid composition. Lysosomal stress has been shown in cultured cells to promote the release of exosomes, a major type of EV [75, 76]. Exosomes are generated when a multivesicular endosome (MVE) fuses with the plasma membrane rather than the lysosome, releasing its intraluminal vesicles into extracellular space [41, 54]. Lysosomal blockade increases the probability that an MVE will fuse with the plasma membrane [75, 76]. If lysosomal stress rather than outright failure is sufficient to trigger increased exosome release, it could account for the overabundance of EVs in *Gba1b* mutants.

A second explanation for increased EVs in GCase-deficient animals is that abnormal membrane lipid composition may directly alter EV biogenesis. Lipid composition determines membrane fluidity and curvature, and thus controls the size, shape, and fusion kinetics of EVs [77–79]. In fact, lipid rafts, particularly those enriched in ceramide, are required for formation at least one type of EV [78]. Membrane changes such as those caused by GCase deficiency, including accumulation of glucosylceramide and altered ceramide levels [80, 81], could alter EV functioning at any stage from formation to internalization by a recipient cell. Either increased or decreased probability of ceramide-dependent EV formation could lead to increased overall EV production, as suppression of one type of EV has been shown to cause overproduction of another type [82].

While understanding the mechanism by which GCase deficiency causes increased EV release is an important goal of future work, an equally important question is how increased EV abundance in *Gba1b* mutants promotes the accumulation of protein aggregates. EVs have been increasingly implicated in the pathogenesis of neurodegenerative disease. Many disease-associated proteins, including prion protein, α-synuclein, β-amyloid, and tau, are detected in EVs [41, 83, 84], which have been proposed as vehicles for the well-documented progressive spread of protein aggregates from one brain region to another [83, 85, 86]. In support of this model, toxic forms of these disease-associated proteins are more abundant in EVs from humans with neurodegenerative diseases such as Alzheimer disease, dementia with Lewy bodies, and Parkinson disease (PD) [84, 87, 88], and EVs from these patients can induce protein aggregation in recipient cells under experimental conditions [89, 90]. However, progression of these diseases has not yet been conclusively demonstrated to be mediated by EVs. Perhaps the strongest evidence that EVs promote the spread of protein aggregates has been found for prion protein. Stimulating the release of EVs increased the cell-to-cell spread of misfolded prion protein, and decreasing EV release reduced the spread [91]. Our findings appear to follow the same pattern: genetic interference with EV production suppressed protein aggregation in *Gba1b* mutants. If the same holds true for other aggregation-prone proteins, conditions that increase EV release could promote the spread of protein aggregates and thus be risk factors for neurodegenerative disease.

**Fig 11:**
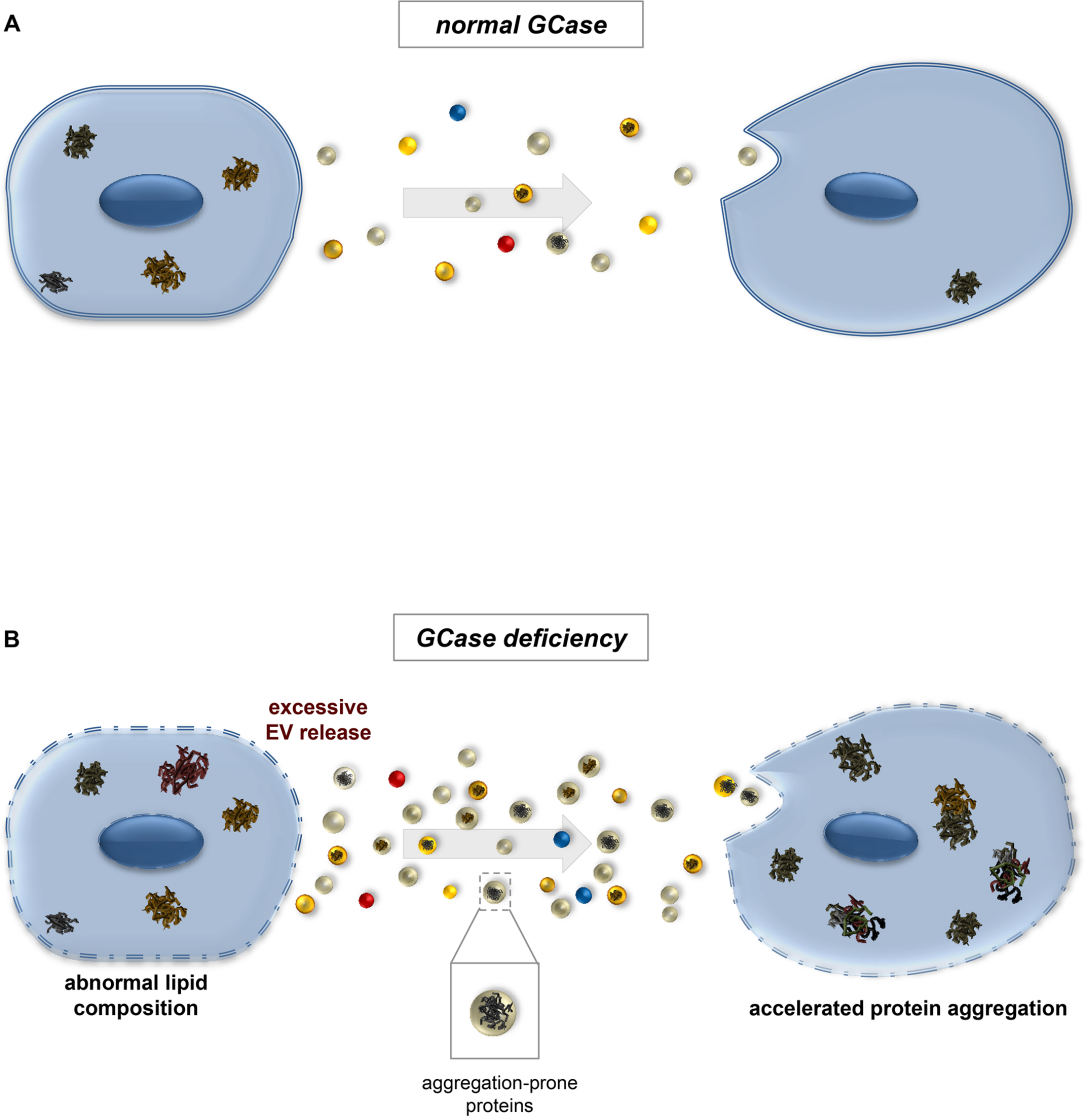
Model: Increased EV production promotes the spread of protein aggregates in glucocerebrosidase-deficient organisms. The diagram shows a possible mechanism by which altered extracellular vesicle biology in *Gba1b* mutants could lead to excess formation of protein aggregates. (A) Under normal conditions, appropriate numbers and types of extracellular vesicles are produced. In reality, most cells both release and receive EVs; here travel is shown in one direction to illustrate the possibility that some cells have high rates of protein aggregation and act as aggregate donors. Some of these EVs transport aggregate-forming protein “seeds” to recipient cells, but the recipients are able to limit aggregate formation via quality control mechanisms. (B) Without the *Gba1b* gene product glucocerebrosidase (GCase), glucosylceramide accumulates in cellular membranes, causing altered membrane lipid composition. Altered membrane composition leads to increased formation and release of extracellular vesicles. Larger numbers of aggregate-bearing extracellular vesicles are taken up by recipient cells, leading to accelerated protein aggregation.

Fig 11 illustrates this model. When GCase activity is normal (Fig 11A), EVs travel between cells, carrying both factors that promote protein aggregation (e.g., disease-associated proteins such as α-synuclein) [88, 92] and factors that oppose it (e.g., chaperones) [93]. Some cells likely generate more aggregates than others, and may therefore release more aggregate-promoting factors, including small aggregate “seeds.” Quality control mechanisms in recipient cells successfully combat protein aggregation, and aggregates accumulate only slowly with age. If GCase activity is absent or reduced, however (Fig 11B), more EVs are generated; this results in greater cell-to-cell transfer of aggregate-prone proteins, perhaps simply because these proteins are normally part of EV cargo. In particular, they may be normal cargo of ESCRT-dependent EVs, given our finding that knockdown of ESCRTs in *Gba1b* mutants ameliorated the mutants’ protein aggregation phenotype. Alternatively, GCase deficiency may alter cargo selection so that more aggregation-prone proteins are loaded into EVs. The net effect of the EV changes is transfer of aggregation-producing factors in quantities that overwhelm quality control mechanisms, leading to excessive accumulation of ubiquitin-protein aggregates in recipient cells.

*GBA* mutations are the strongest single risk factor for PD and dementia with Lewy bodies, affecting up to 10% of PD patients worldwide [2, 5]. Our finding that GCase deficiency causes increased EV release offers new insight into these prevalent disorders. For example, increased transmission of protein aggregates via EVs could explain the earlier onset and faster disease progression in PD patients with *GBA* mutations [6, 94–97]. Future investigations should determine how glucocerebrosidase deficiency increases EV abundance, and how manipulations of EV production might prevent or delay the progression of neurodegenerative disease.

## Materials and methods

### *Drosophila* strains and culture

Fly stocks were maintained on standard cornmeal-molasses food at 25°C. The *Gba1b* null (*Gba1b*^*ΔTT*^), *Gba1b* control (*Gba1b*^*rv*^), *Atg7*^*d4*^, *Atg7*^*d77*^, *Sod2*^*n283*^, *Sod2*^*wk*^, *park*^*25*^, *PINK1*^*B9*^, *and PINK1*^*rv*^ alleles, as well as the *UAS-PINK1#2* strain, have been previously described [9, 19, 20, 98, 99]. The *UAS-ALiX-HA* strain was obtained from the former Bangalore Fly center (National Centre for Biological Sciences, Bangalore, India). The *UAS-Ref(2)P-RNAi* strain (v108193) was obtained from the Vienna Drosophila Resource Center. Other strains and alleles were obtained from the Bloomington Stock Center: *elav-GAL4* (458), *Act5C-GAL4* (3953), *UAS-Hsc70-4* (5846), *w*^*1118*^ (3605), *UAS-Rab11-GFP* (8506), *Gba1b*^*MB03039*^ (23602) [100], *UAS-Mvb12*-*RNAi* (43152), *UAS-larsen*-*RNAi* (38289) [101], and *UAS-CHMP2B*-*RNAi* (38375) [102]. *Atg7* null mutants were *Atg7*^*d4*^/*Atg7*^*d77*^ transheterozygotes. *Sod2* mutants were null/hypomorph compound heterozygotes (*Sod2*^*n283*^/*Sod2*^*wk*^). The full genotype of *parkin* mutants was *If/CyO; park*^*25*^*/park*^*25*^. The WT controls for *Atg7* and *parkin* mutants were a composite dataset derived from four groups of healthy flies with intentionally diverse genetic backgrounds (see protein turnover rate calculations section). The control for *PINK1*^*B9*^ was its revertant (precise excision) strain, *PINK1*^*rv*^, and the control for *Sod2* was *CyO/+*. The control strain for *Gba1b* was the revertant *Gba1b*^*rv*^. In Fig 7 we used the following genotypes for the experiments involving the *ALiX-HA* transgene: control = *Gba1b*^*rv*^*/Gba1b*^*MB03039*^; *Gba1b* = *Gba1b*^*ΔTT*^*/Gba1b*^*MB03039*^. This combination of *Gba1b* mutant alleles, which we used for ease of recombination with the *ALiX-HA* transgene, produced the same biochemical abnormalities found in *Gba1b*^*ΔTT*^ homozygotes (S5 Fig).

### Targeted lipidomics

Lipidomic analysis was performed at the Northwest Metabolomics Research Center at the University of Washington, Heads were isolated from 10-day-old control and *Gba1b* flies flash-frozen in liquid nitrogen, and lipids were then extracted from the frozen head tissue. Levels of glucosylceramide and ceramide were measured by a high-performance liquid chromatography/mass spectrometry (LC-MS/MS) method, using a sphingolipids mix as internal standard (Avanti Sphingolipids Mix II LM-6005). Results were expressed as lipid levels per mass of starting tissue. For each lipid species, three independent samples were analyzed.

### Preparation of labeled food

[5,5,5 – ^2^H_3_] leucine (D3-leucine; 99 atom % deuterium) was obtained from Isotec/Sigma-Aldrich. Synthetic complete medium without leucine (C-Leu) was supplemented with glucose and 60 mg/L D3-leucine. A strain of *Saccharomyces cerevisiae* auxotrophic for leucine (BB14-3A, Brewer Lab, University of Washington [103]) was grown to saturation at 30°C, then spun down, flash-frozen in liquid nitrogen, lyophilized, and stored at −80°C.

Because brewing in-house produced limited quantities of labeled yeast, we made labeled fly food in batches of ~40 mL using a microwave. We did this by substituting cornstarch for cornmeal in the lab’s standard recipe (2.35% yeast w/v) and dispensing the cooked food in small amounts into vials lined with wet Whatman paper to maintain moisture. Unlabeled transition food for the first 24 hours after eclosion was made and dispensed in the same way, substituting Red Star yeast.

### *In vivo* stable isotope labeling of flies

#### *Atg7*, *parkin*, *PINK1*, and *Sod2*

These mutants and their controls were labeled using D3-leucine yeast paste as previously described [21].

#### Gba1b

Groups of 20–30 male *Gba1b* (*GBA1*^*ΔTT*^) or *GBA1*^*rv*^ flies were selected on the day of eclosion and provided with unlabeled transition food for 24 h. They were then given food made with D3-leucine–labeled yeast and were maintained in humidified containers at 25°C, with food replaced every two days. After 120 h or 264 h of labeling, flies were flash-frozen in liquid nitrogen. Three biological replicates (~50 heads each) were obtained for each genotype and time point.

#### Old vs. young

Groups of 20–30 male *w*^*1118*^ flies received labeled food starting at 5 days or 55–60 days of age. Flies were frozen 120 or 240 h after the start of labeling. Labeling was otherwise performed as in the *Gba1b* study.

### Mass spectrometry sample preparation

#### *Gba1b* and old vs. young studies

Frozen flies were vortexed to remove heads, and the isolated heads were homogenized in 0.1% RapiGest solution in 50 mM ammonium bicarbonate (Waters Corporation, 186001861) using a 0.2-mL Wheaton micro tissue grinder (Fisher Scientific, 08-414-15B). Homogenates were centrifuged at 4°C at 1600 x *g* for 10 min, and then at 6500 x *g* for 10 min, to remove debris and nuclei. The supernatants were then incubated with DTT (final concentration 5 mM) at 60°C for 30 min. Iodoacetamide was added to a final concentration of 15 mM, and the samples were incubated at room temperature in the dark for 30 min. Trypsin (Fisher Scientific, PR-V5111) was added at a ratio of 1 μg trypsin per 50 μg protein, and incubated for 1 h at 37°C with shaking. RapiGest was hydrolyzed by adding HCl to a final concentration of 200 mM, followed by incubation at 37°C with shaking for 45 min. The samples were then centrifuged for 10 min at 4°C at 20,000 x *g*, and the supernatant was collected.

#### *Atg7*, *parkin*, *PINK1*, and *Sod2*

Samples were prepared as described above except that supernatants were boiled 7 min before incubation with DTT.

### Liquid chromatography and mass spectrometry

*Atg7*, *parkin*, *PINK1*, and *Sod2* mutant samples were processed as previously described [21]. *GBA1b* and old/young samples were processed as follows: Fused silica microcapillary columns of 75 μm inner diameter (Polymicro Technologies, Phoenix, AZ) were packed in-house by pressure loading 30 cm of Jupiter 90 Å C12 material (Phenomenex). Kasil (PQ Corporation) frit microcapillary column traps of 100 μm inner diameter with a 2-mm Kasil frit were packed with 4 cm of Jupiter 90 Å C12. A retention time calibration mixture (Pierce) was used to assess quality of the column before and during analysis. Three of these quality control runs were analyzed prior to any sample analysis, and another quality control run was performed after every six sample runs. One microgram of each sample digest and 150 femtomoles of the quality control sample were loaded onto the trap and column by the NanoACQUITY UPLC system (Waters Corporation). Buffer solutions used were water, 0.1% formic acid (buffer A), and acetonitrile, 0.1% formic acid (buffer B). The 60-minute gradient of the quality control consisted of 30 minutes of 98% buffer A and 2% buffer B, 5 minutes of 65% buffer A and 35% buffer B, 10 minutes of 40% buffer A and 60% buffer B, 5 minutes of 95% buffer A and 5% buffer B, and 18 minutes of 98% buffer A and 2% buffer B at a flow rate of 0.3 μL/min. The 240-minute gradient for the sample digest consisted of 120 minutes of 98% buffer A and 2% buffer B, 80 minutes of 65% buffer A and 35% buffer B, 20 minutes of 20% buffer A and 80% buffer B, and 20 minutes of 98% buffer A and 2% buffer B at a flow rate of 0.25 μL/min. Peptides were eluted from the column and electrosprayed directly into an Q-Exactive HF mass spectrometer (Thermo Fisher) with the application of a distal 3 kV spray voltage. For the quality control analysis, a cycle of one 60,000 resolution full-scan mass spectrum (400–1600 *m/z*) was followed by 17 data-independent MS/MS spectra using an inclusion list at 15,000 resolution, 27% normalized collision energy with a 2 *m/z* isolation window. For the sample digests, a cycle of one 120,000 resolution full-scan mass spectrum (400–1600 *m/z*) followed by 20 data-dependent MS/MS spectra on the top 20 most intense precursor ions at 15,000 resolution, 27% normalized collision energy with a 1.5 *m/z* isolation window. Application of the mass spectrometer and UPLC solvent gradients was controlled by the Thermo Fisher XCalibur data system.

### Analysis of mass spectrometry data

The quality control sample data were analyzed using Skyline [23]. High-resolution MS data were processed by BullsEye to optimize precursor mass information [22]. The MS/MS output was searched using COMET [104] with differential modification search of 3.0188325 Da for leucine and 15.994915 methionine and a static modification of 57.021461 Da for cysteine, against a FASTA database containing all the protein sequences from FlyBase plus contaminant proteins. Peptide-spectrum match false discovery rates were determined using Percolator [105] at a threshold of 0.01, and peptides were assembled into protein identifications using an in-house implementation of IDPicker [106].

### Calculation of protein turnover and abundance

Turnover rates were calculated using Topograph software [22]. For a full description of Topograph settings, see Vincow et al. [21]. A protein’s turnover rate was computed based on data from all peptides detected, and values from all biological replicates were pooled for turnover calculations. A protein’s turnover rate was calculated based on at least 6 measurements per genotype of percent turnover for *GBA1b* mutants and old/young flies, and at least 15 measurements per genotype for *Atg7*, *parkin*, *PINK1*, or *Sod2* mutants. Peptides that could be the product of more than one gene were excluded from analysis. For a small percentage of genes (2%-5%), Topograph clustered peptides corresponding to a single gene into 2-3 nonoverlapping “isoform groups.” For example, isoform group 1 might include peptides mapping only to the COX6B-PA isoform, while isoform group 2 peptides could have come from COX6B-PA, -PB, or -PC. While in most cases the isoform groups for a single protein had essentially identical turnover rates, occasionally they displayed significant differences in turnover behavior. Each isoform group was therefore analyzed as a separate protein.

We excluded proteins with excessive inter-replicate variability of turnover rates, defined as coefficient of variation ≥ 0.25. We calculated the turnover rate separately for each biological replicate and determined the coefficient of variation across replicates. Proteins were analyzed only if they met inclusion criteria in both mutants and controls.

In previous work, we had compared *Atg7* and *parkin* null mutants to their respective heterozygotes [21]. However, we later found that both *Atg7* and *parkin* heterozygotes had mild but significant slowing of mitochondrial protein turnover compared to WT flies, and we selected the WT dataset as a more appropriate control. For turnover analyses, *Atg7* and *parkin* nulls were both compared to a composite WT dataset derived from four separate groups of healthy flies (*w*^*1118*^, *PINK1*^*rv*^, *CyOActGFP/+*, and a mixture of`*CyO/Hsp70-GAL4* and *CyO/UAS-PINK1#2*). Turnover rates are the mean values for all genotypes in which the protein was detected; the rates are highly consistent across genotypes, as previously reported [21]. Each mean value for a genotype was treated as one replicate for statistical purposes. Statistical significance of fold change in turnover was calculated for groups of proteins using nested ANOVA [107], and significance of change for individual proteins was calculated using *t* tests. The following subgroups of proteins had enough replicates for *t* tests: 148 mitochondrial, 36 ribosomal, 15 ER/peroxisomal, and 275 nonorganellar proteins.

We measured protein abundance from the same raw mass spectrometry data used in the turnover study, using Skyline [23] and MSstats [24]. Prior to MSstats analysis, we obtained total abundance (labeled plus unlabeled) for each peptide using a custom R script. The statistical significance of intergroup differences was calculated using a linear mixed model, then adjusted for multiple comparisons by the Benjamini-Hochberg procedure with a false discovery rate of 0.05. All abundance comparisons were made at the second time point, when differences between genotypes were most marked. In abundance analyses, *parkin* and *Atg7* mutants were compared to their original heterozygote controls rather than WT flies (see calculations above). While the composite control group approach was appropriate for measurement of turnover, which is more consistent and less noisy than abundance [21], measurement of relative protein abundance required mutant and control samples that had been run at the same time.

### Annotation and classification of *Drosophila* proteins

*General*: *Drosophila* protein localization was determined from a variety of resources including gene and protein information databases (FlyBase [108], MitoDrome [109], NCBI [110], UniProt [111]), protein localization prediction algorithms (WoLF PSORT [112], MitoProt [113], Predotar [114], SignalP [115], NucPred [116], and PTS1 Predictor [117, 118]), BLAST [119], and primary literature.

*Proteasome substrates*: We identified proteins as proteasome substrates (Fig 4) if their mammalian orthologs had one or more regulated ubiquitinated sites according to Wagner et al. [35]. These sites showed altered abundance of ubiquitinated peptides after proteasome inhibitor treatment. We identified *Drosophila* orthologs of proteins from the Wagner et al. data with the DRSC Integrative Ortholog Prediction Tool (DIOPT) v6 [50], minimum score 5.

*Microautophagy substrates*: We identified microautophagy substrates by searching for targeting sequences (Fig 4), also called KFERQ-like motifs. These motifs were defined as sequences of five amino acids (AAs) that fit criteria established by Dice [120, 121]:

1. The sequence begins or ends with Q.
2. The sequence contains either one or two basic AAs (K, R), one or two bulky hydrophobic AAs (F, I, L, V), and one acidic AA (D, E).

We wrote an algorithm using Python 2.7 to search protein sequences for these motifs and applied it to the fly proteome (FASTA sequences downloaded from FlyBase). We then identified cytosolic proteins by annotation as described above, and compared the effects of GCase deficiency on cytosolic proteins with and without KFERQ-like sequences.

*Endocytic turnover substrates*: Proteins designated endocytic turnover substrates in Fig 5 were identified using FlyBase annotation and search terms such as *receptor*, *transmembrane*, *extracellular matrix*, *integral component of plasma membrane*, and *channel*. Endosomal machinery proteins (see below) were excluded.

*Endosomal machinery*: Proteins designated “endosomal machinery” in Fig 5 were identified by a FlyBase search for the string “endosom*” in at least one of the following fields: GO Molecular Function, GO Biological Process, GO Cellular Component, Gene Snapshot, or UniProt Function.

*Extracellular vesicle proteins*: To identify extracellular vesicle proteins, we compiled a list of proteins detected in EVs in mass spectrometry studies of *Drosophila* cultured cells [46–49]. The list contained 544 unique proteins, 329 of which were found in *Gba1b* mutant protein turnover data and 499 in abundance data. In addition, we obtained from ExoCarta [47] the list of “top 100 [mammalian] proteins that are often identified in exosomes,” and identified 97 *Drosophila* orthologs of these proteins using DIOPT v6 as previously described [50]. Fifty-nine proteins from this list were found in *Gba1b* mutant turnover data and 86 in abundance data.

The significance of intergroup differences was evaluated using the Fisher exact test except when the total number of proteins was too large, in which case we performed a χ^2^ test of homogeneity.

### Proteasome enzyme activity assay

Proteasome activity was measured in heads from male and female flies 10 to 11 days old (50 per sample) according to the method of Tsakiri et al. [122], with the following modifications: We used 26S lysis buffer only. We obtained substrate buffer and fluorescent substrates from the UBPBio Proteasome Activity Fluorometric Assay Kit II (J4120), and we used epoxomicin 20 μM for proteasome inhibition. Specifically, we divided the lysate in half and added DMSO to one half and epoxomicin to the other. We measured the protein concentration of lysates using the Pierce BCA Protein Assay Kit (23227), and measured sample fluorescence with a Synergy H1 BioTek plate reader (excitation 350 nm, emission 450 nm). We subtracted the activity measured in the epoxomicin-treated homogenate from the activity in the DMSO-treated homogenate. The experiment was repeated three times.

### Extraction of hemolymph and preparation of EV fractions

For total EVs (tEVs), hemolymph was obtained from 20 flies (10 males and 10 females, 10 to 11 days old) per sample. In order to obtain whole-fly homogenate from the same animals used for collection of hemolymph, hemolymph was extracted manually from the first four flies and their bodies were reserved for later use. These flies were decapitated, following which their hemolymph was collected by pressing on the thorax with the head of a butterfly pin. The hemolymph from these four flies was collected by capillary action into 1 μL PBS, and the total sample was transferred to a 1.7-mL microfuge tube containing 9 μL PBS. The heads and bodies of the four flies were then homogenized in RIPA buffer for whole-fly protein homogenates. Two holes were made with a 25-g needle in the bottom of a 0.5-mL tube, and 16 more flies were decapitated and placed in this tube. The 0.5-mL tube was then seated in the PBS-containing 1.7-mL tube for centrifugation. The tubes were centrifuged at 5000 x *g* for 5 min at 4°C, after which the extracted hemolymph was centrifuged for 30 min at 10,000 x *g* at 4°C to remove cell debris and the cell-free supernatant was collected. An equal volume of 2x Laemmli buffer (4% SDS, 20% glycerol, 120 mM Tris-Cl pH 6.8, 0.02% bromophenol blue, 2% β-mercaptoethanol) was added to the cell-free supernatant and also to the whole-fly protein homogenates, and all samples were boiled for 10 min and then stored at −80°C. The experiment was repeated at least three times.

Small EVs (sEVs) were prepared as for tEVs, with the following changes: 50–60 adult flies were used per sample. The hemolymph was collected into a volume of PBS scaled to the number of flies used (1 μL/fly) to minimize sample loss during filtration. After the 10,000 x *g* spin, Total Exosome Isolation Reagent for Cell Culture (Thermo Fisher/Invitrogen, 4478359) was used as in Tassetto et al. [123] except that we used Ultrafree 0.22 μm spin filters (Fisher, UFC30GV0S). The resulting filtrate was boiled and stored as for the tEVs.

### Preparation of Triton-soluble and insoluble fractions

Heads from 10-day-old flies (6 females and 6 males per sample) were homogenized in Triton lysis buffer (50 mM Tris-HCl pH 7.4, 1% Triton X-100, 150 mM NaCl, 1 mM EDTA), and then spun at 15,000 x *g* for 20 min. The detergent-soluble supernatant was collected and mixed with an equal volume of 2x Laemmli buffer, and the same buffer was used to resuspend the Triton-insoluble pellet. All samples were boiled for 10 minutes. The Triton-insoluble protein extracts were then cleared of debris by centrifugation at 15,000 x *g* for 10 minutes, followed by collection of the supernatant. At least three independent experiments were performed.

### Western blotting

Proteins were separated by SDS-PAGE on 4%-20% MOPS-acrylamide gels (GenScript Express Plus, M42012) and electrophoretically transferred onto Immobilon PVDF membranes (Fisher, IPVH00010). Immunodetection was performed using the iBind Flex Western Device (Thermo Fisher, SLF2000). Antibodies were used at the following concentrations: 1:25,000 mouse anti-Actin (Chemicon/Bioscience Research Reagents, MAB1501), 1:250 mouse anti-Rab11 (BD Transduction Laboratories, 610657), 1:200 rabbit anti-Ref(2)P (Abcam, ab178440), 1:500 mouse anti-ubiquitin (Santa Cruz, sc-8017), 1:800 mouse anti-Cnx99A (DHSB, Cnx99A 6-2-1), 1:100 mouse anti-Golgin-84 (DHSB, Golgin84 12–1), and 1:500 rat anti-HA (Sigma-Aldrich, 11867423001). HRP secondary antibodies were used as follows: 1:500 to 1:1000 anti-mouse (BioRad, 170–6516), 1:100 anti-rat (Sigma-Aldrich, A9037), and 1:500 to 1:1000 anti-rabbit (BioRad, 172–1019). Signal was detected using Pierce ECL Western Blotting Substrate (Fisher, 32106). Densitometry measurements of the western blot images were measured blind to genotype and condition using Fiji software [49]. For homogenates, signal was normalized either to Actin or to Ponceau-S [124, 125]. For EVs, signal was normalized to loading volume. Normalized western blot data were log-transformed when necessary to stabilize variance before means were compared using Student *t* test. Each experiment was performed at least three times.

### Nanoparticle tracking analysis

EVs were prepared for nanoparticle tracking analysis (NTA) as described for western blotting through the 10,000 x *g* step, after which they were passed through a 0.65 μm Ultrafree-MC filter (Fisher, UFC30DV0S) to ensure removal of any remaining cellular debris and stored at −80°C. Hemolymph was obtained from 60 flies per sample, and four biological replicates per genotype were collected.

EV size and concentration were measured using NTA by Alpha Nano Tech LLC (Research Triangle Park, NC). NTA was performed using a ZetaView instrument equipped with an sCMOS camera and 532 nm laser. Instrument parameters were as follows: temperature setting 23°C, Max Area 500, Min Area 20, Min Brightness 20. Two cycles of analysis at 11 positions were performed for each sample. Data were analyzed using ZetaView software version 8.04.02.

Standard laboratory protection equipment was used during all steps of sample preparation and analysis to prevent sample contamination with dust particles. The 1x PBS solution (Amresco) used to dilute samples was filtered on the day of analysis through a 0.22 μm Millex-GV syringe filter (Millipore), and its purity was confirmed by NTA analysis prior to the study. Instrument qualification was performed by analyzing a polystyrene bead standard (100 nm, Particle Metrix) in 1x PBS prior to each study. Instrument accuracy and precision were confirmed to ± 5% of the target value.

## Supporting information

S1 Fig *Gba1b* mutants have markedly increased glucosylceramide and moderately decreased ceramide. Targeted lipidomics on fly heads from *Gba1b* mutants and controls (*n* = 3 biological replicates). Error bars represent SD. (A) Levels of glucosylceramide (GluCer) relative to internal standards, per milligram of head protein. (B) Levels of 12:0 ceramide (Cer) relative to internal standards, per milligram of head protein. *p < 0.05 by Student *t* test.
(TIF)

S2 Fig EV-associated and non-EV proteins with significantly faster turnover in *parkin*, *PINK1*, and *Sod2* mutants. EV-associated proteins do not have an increased prevalence of faster turnover in any of the three mutants compared to their respective controls (*p* > 0.05 by Fisher exact test). All measurements were performed on fly head extracts.
(TIF)

S3 Fig Exogenously expressed Rab11, like native Rab11, is more abundant in EVs from *Gba1b* mutants compared to controls. Pan-neuronal driver *elav-GAL4* was used to express Rab11-GFP in *Gba1b* mutants and controls. Whole-fly homogenates and isolated small extracellular vesicles (sEVs; see Materials and Methods) were probed with an antibody to Rab11. (A) Representative image of western blot showing native (arrow) and GFP-tagged (arrowhead) Rab11. (B) Quantification of Rab11-GFP. At least three independent experiments were performed. Error bars represent SEM. *p < 0.05.
(TIF)

S4 Fig Knockdown of Ref(2)P confirms the identity of high molecular weight forms of the protein. The ubiquitous *Act5C-GAL4* driver was used to express RNAi against *Ref(2)P* in *Gba1b* mutants. Whole-body homogenates were probed with an antiserum to Ref(2)P. A representative blot is shown. Loading control is Ponceau-S staining. At least three independent experiments were performed.
(TIF)

S5 Fig The *Minos* insertion allele of *Gba1b* has the same biochemical phenotypes as the deletion allele. (A) Representative image and quantification of insoluble ubiquitinated protein. Western blotting was performed on Triton-insoluble fractions from heads of 10-day-old control flies (*Gba1b*^*rv*^*/Gba1b*^*MB03039*^) and *Gba1b* mutants (*Gba1b*^*ΔTT*^*/Gba1b*^*MB03039*^). (B) Representative image and quantification of soluble Ref(2)P. Western blotting was performed on Triton-soluble fractions of *Gba1b* and control flies (as above). Experiments were performed at least three times. *p < 0.05 by Student *t* test.
(TIF)

S1 Data Protein turnover and abundance data for *Gba1b* mutants, *Atg7* mutants, *parkin* mutants, *PINK1*^*B9*^ mutants, and *Sod2* mutants vs. their respective controls. (XLSX)

S2 Data Lists of cytosolic proteasome substrates, cytosolic proteins with KFERQ motifs, endocytic turnover substrates, and endosomal machinery proteins. (XLSX)

S3 Data Two lists of EV-associated proteins: Proteins detected in EVs from *Drosophila* cultured cells, and *Drosophila* orthologs of the ExoCarta mammalian “top 100” list. (XLSX)

S4 Data Protein turnover and abundance data for old vs. young WT flies. (XLSX)
