# Supplementary figures and images for: Glucocerebrosidase deficiency promotes protein aggregation through dysregulation of extracellular vesicles
Source: PLoS Genet. 2018 Sep 26;14(9):e1007694. doi: 10.1371/journal.pgen.1007694 (PMC6175534; doi:10.1371/journal.pgen.1007694)

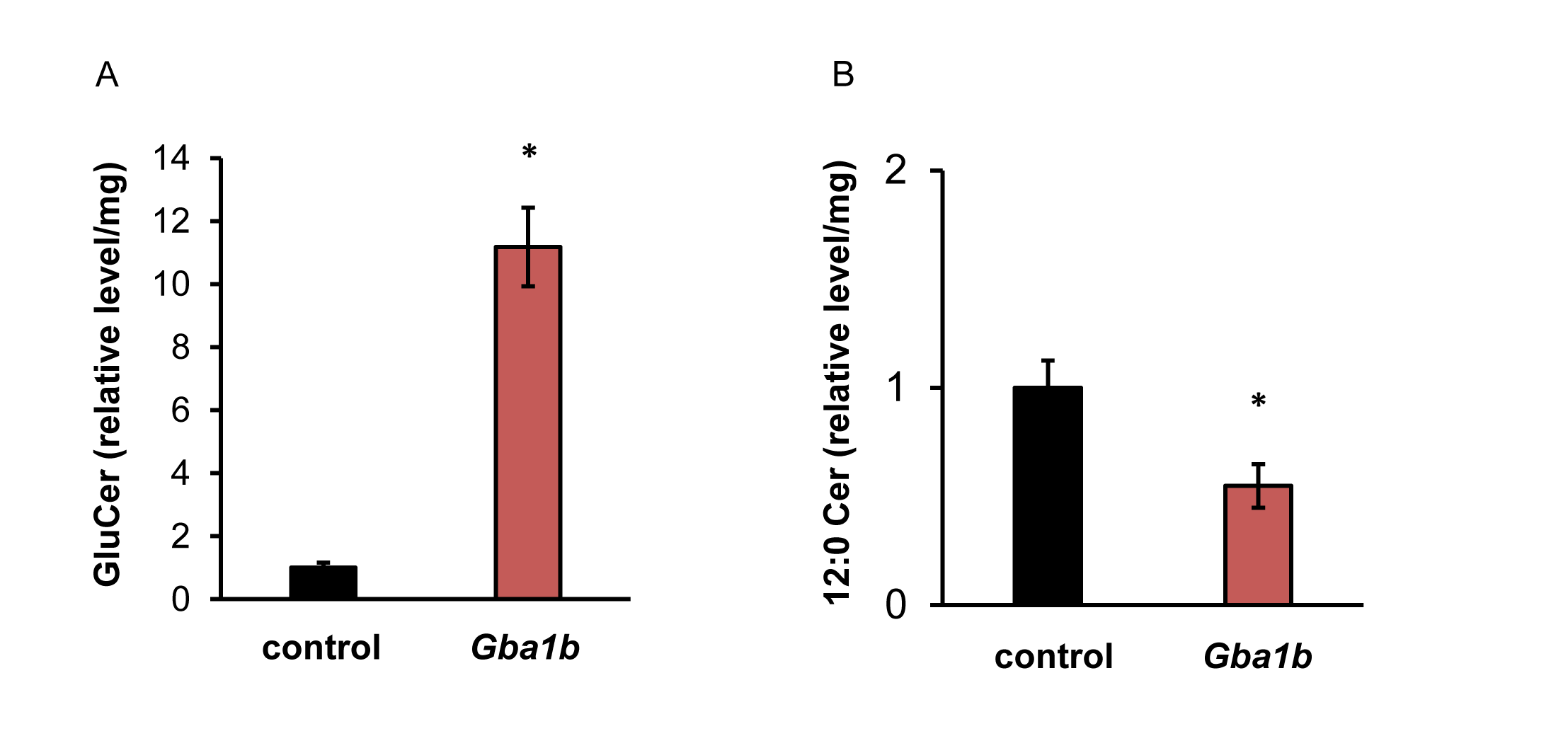

Supplement: S1 Fig — Targeted lipidomics on fly heads from Gba1b mutants and controls (n = 3 biological replicates). Error bars represent SD. (A) Levels of glucosylceramide (GluCer) relative to internal standards, per milligram of head protein. (B) Levels of 12:0 ceramide (Cer) relative to internal standards, per milligram of head protein. *p < 0.05 by Student t test. (TIF) [file pgen.1007694.s001.tif]

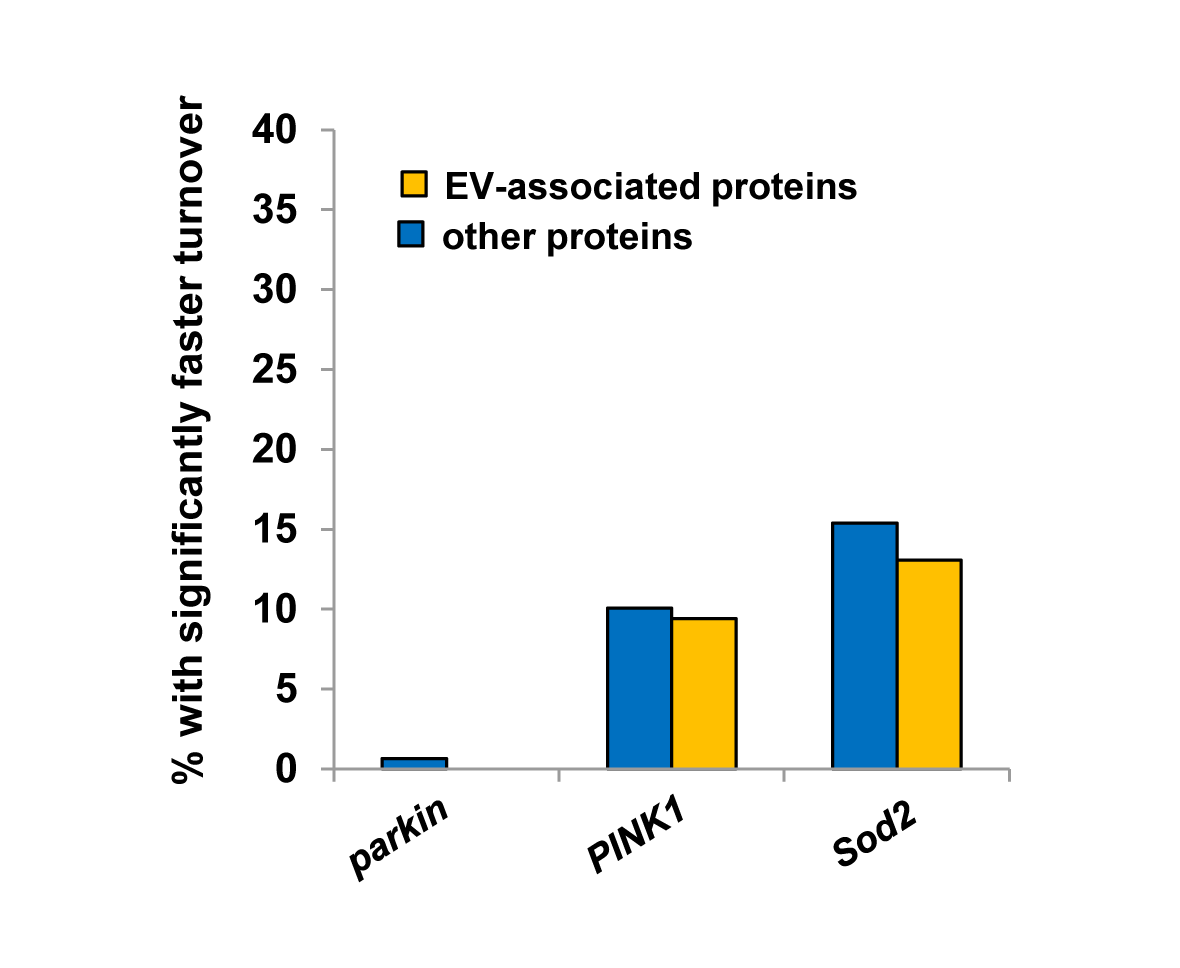

Supplement: S2 Fig — EV-associated proteins do not have an increased prevalence of faster turnover in any of the three mutants compared to their respective controls (p > 0.05 by Fisher exact test). All measurements were performed on fly head extracts. (TIF) [file pgen.1007694.s002.tif]

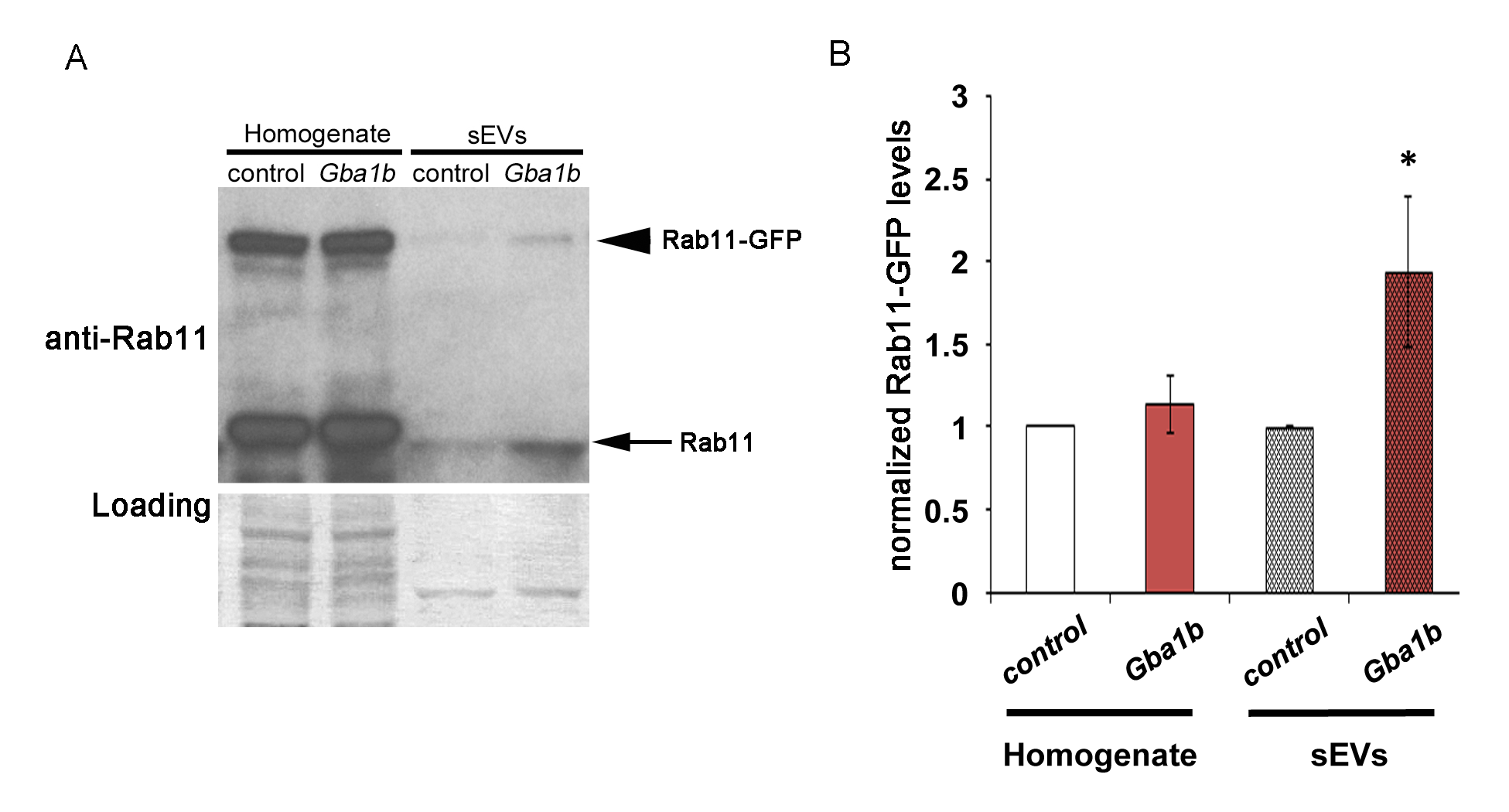

Supplement: S3 Fig — Pan-neuronal driver elav-GAL4 was used to express Rab11-GFP in Gba1b mutants and controls. Whole-fly homogenates and isolated small extracellular vesicles (sEVs; see Materials and Methods) were probed with an antibody to Rab11. (A) Representative image of western blot showing native (arrow) and GFP-tagged (arrowhead) Rab11. (B) Quantification of Rab11-GFP. At least three independent experiments were performed. Error bars represent SEM. *p < 0.05. (TIF) [file pgen.1007694.s003.tif]

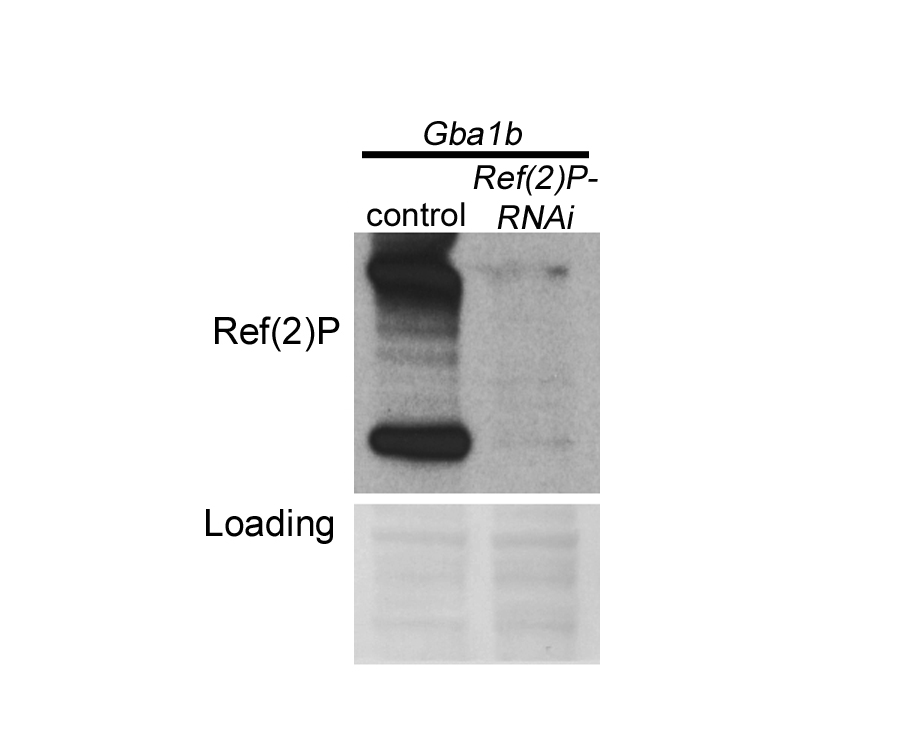

Supplement: S4 Fig — The ubiquitous Act5C-GAL4 driver was used to express RNAi against Ref(2)P in Gba1b mutants. Whole-body homogenates were probed with an antiserum to Ref(2)P. A representative blot is shown. Loading control is Ponceau-S staining. At least three independent experiments were performed. (TIF) [file pgen.1007694.s004.tif]

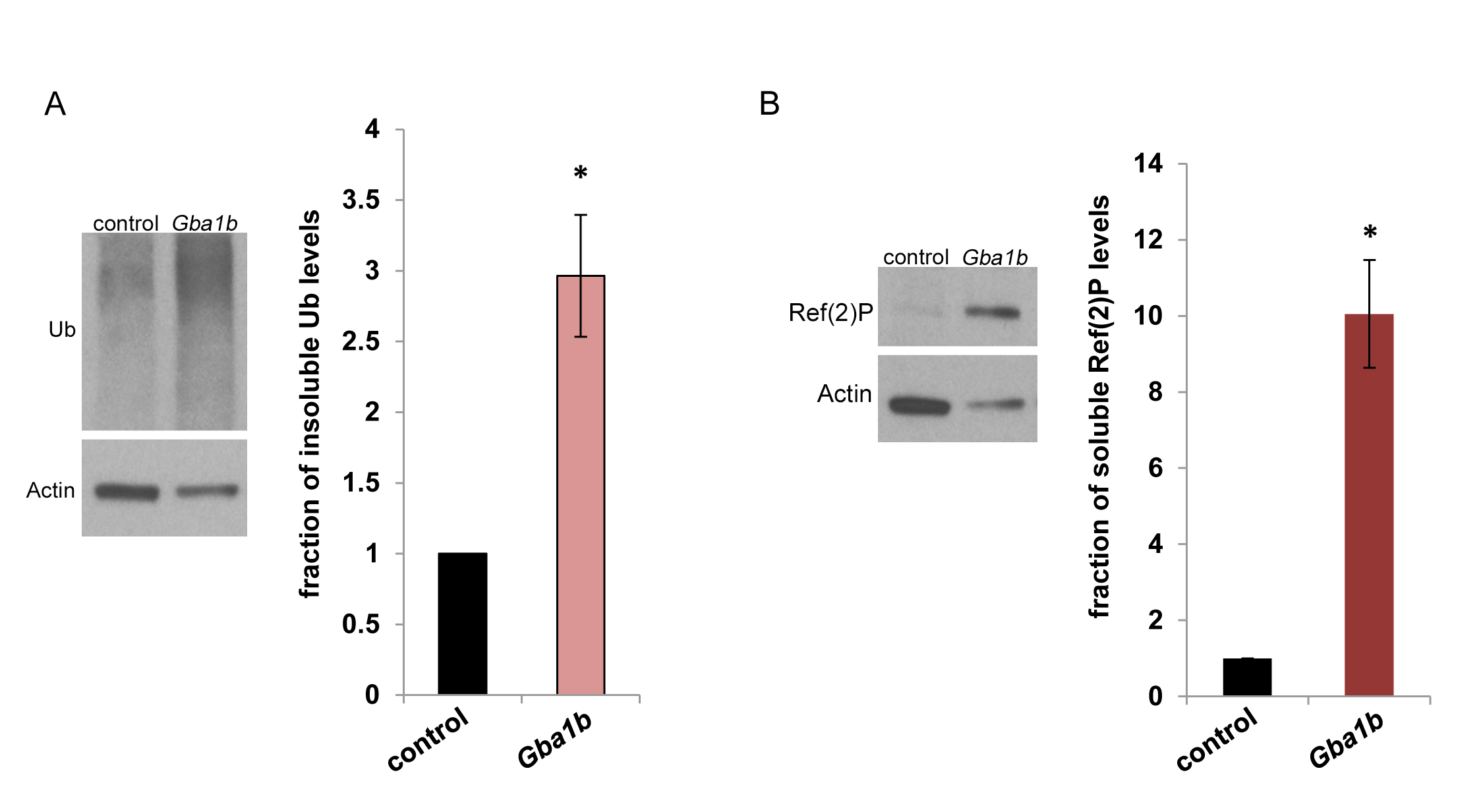

Supplement: S5 Fig — (A) Representative image and quantification of insoluble ubiquitinated protein. Western blotting was performed on Triton-insoluble fractions from heads of 10-day-old control flies (Gba1brv/Gba1bMB03039) and Gba1b mutants (Gba1bΔTT/Gba1bMB03039). (B) Representative image and quantification of soluble Ref(2)P. Western blotting was performed on Triton-soluble fractions of Gba1b and control flies (as above). Experiments were performed at least three times. *p < 0.05 by Student t test. (TIF) [file pgen.1007694.s005.tif]
